# Future heatwave conditions inhibit CO_2_ ‐induced stomatal closure in wheat

**DOI:** 10.1111/nph.70722

**Published:** 2025-11-16

**Authors:** Robert S. Caine, Muhammad S. Khan, Yixiang Shan, Colin P. Osborne, Holly L. Croft

**Affiliations:** ^1^ Plants, Photosynthesis and Soil, School of Biosciences University of Sheffield Sheffield South Yorkshire S10 2TN UK; ^2^ Institute for Sustainable Food, School of Biosciences University of Sheffield Sheffield South Yorkshire S10 2TN UK

**Keywords:** climate change, heatwaves, high CO_2_, high VPD, nitrogen, stomata, transpiration, wheat

## Abstract

- As global temperatures and the severity of droughts continue to increase, food crops will more frequently experience high vapour pressure deficit (VPD) during heatwave events. However, the interactive effects of rising atmospheric CO_2_ and high‐VPD heatwaves on crop water fluxes and yields are currently unknown.
- We investigate stomatal, photosynthetic and productivity changes in wheat during simulated future high‐VPD heatwaves, under ambient (450 ppm) or elevated (720 ppm) CO_2_ concentrations, across four N‐fertiliser treatments. We measured the physiological response of abaxial and adaxial leaf surfaces to elevated CO_2_ concentration and/or high‐VPD heatwave exposure, and quantified drought responses, seasonal water usage and ear weight.
- Transpiration (*E*) and stomatal conductance (*g*_sw_) increased during high‐VPD heatwaves (irrespective of CO_2_ concentration or N‐fertiliser), largely due to increased water fluxes from abaxial leaf surfaces. Higher *E* and water usage increased wheat vulnerability to drought and led to reduced total ear weight. High‐VPD heatwaves also hindered stomatal responses to light, with *g*_sw_ only reducing by 37–38% after 1 h of dark treatment.
- Our results show that wheat stomata are inhibited from closing under future high CO_2_, high‐VPD heatwave conditions. This has considerable implications for future wheat water requirements, which in‐turn could significantly impact drought susceptibility and yield potential.

## Introduction

The global atmospheric CO_2_ concentration is currently 425 ppm (Keeling *et al*., 1976), having risen from *c*. 280 ppm during pre‐industrial times (Peters *et al*., 2007; Murphy, 2024). Forecasts suggest that CO_2_ increases will continue throughout the 21^st^ century, with Shared Socioeconomic Pathways (SSP) predictions ranging from 393 ppm (SSP1‐1.9) to 1195 ppm (SSP5‐8.5) by 2100 (Meinshausen *et al*., 2020; Lee *et al*., 2023). Without a unified global stance on climate action, it is unclear which SSP is most likely to unfold, with CO_2_ levels of 620–860 ppm possible by the end of the century (Meinshausen *et al*., 2020; Parker & Mainelli, 2024). Many plants benefit from increased CO_2_ concentrations due to enhanced photosynthesis (*A*) and reduced stomatal conductance (*g*_sw_), which promotes greater intrinsic water‐use efficiency (iWUE; *A*/*g*_sw_) and drought tolerance (Ainsworth & Rogers, 2007; Leakey *et al*., 2009). However, with increasing atmospheric CO_2_ expected to co‐occur with rising temperatures and higher VPD, it is unclear what the net impact will be on plant gas exchange, particularly because *E* is fundamental both for evaporative cooling (Bertolino *et al*., 2019) and for obtaining additional nutrients required to support higher rates of growth (Matimati *et al*., 2014; Houshmandfar *et al*., 2018).

Wheat provides 20% of daily calories world‐wide and is the largest crop globally by cultivated land area (Shiferaw *et al*., 2013; Bentley *et al*., 2022; FAOSTAT, 2022). Yields of wheat have increased substantially over the last century, thanks in large part to increased application of inorganic nitrogen (N) fertiliser (Plett *et al*., 2020; Walling & Vaneeckhaute, 2020), but this has been at the expense of increased water usage (Noor *et al*., 2023), with highly fertilised wheat crops considerably more susceptible to drought (Van Herwaarden *et al*., 1998; Caine *et al*., 2024). Increased water usage associated with increased N application is particularly problematic in many low‐income countries, including many regions in Africa, as water availability is often limited due to insufficient rainfall and suboptimal irrigation technologies (Tadesse *et al*., 2018; Negeri & Xiuguang, 2025). With N‐fertiliser also limited in many of the same regions (Silva *et al*., 2023), it will be especially difficult to maximise wheat yields under future conditions in which evaporative demands are forecast to increase (Reda *et al*., 2025). It is critical therefore that we understand how water availability, N application, rising CO_2_ concentration and rising temperatures and VPD impact wheat productivity if we are to foresee how crops will grow under the suboptimal conditions forecast for many areas, particularly those in the Global South.

As with increasing N application, extreme heatwave events around the world also raise wheat water requirements (Shew *et al*., 2020), often resulting in drought and reduced seed set as temperatures become increasingly severe (Djanaguiraman *et al*., 2020; Lorite *et al*., 2023). Currently, drought and heat‐related losses account for 12.4% of global wheat yields (Matiu *et al*., 2017; Bezner Kerr *et al*., 2022), but this yield gap is expected to expand as climate conditions become increasingly extreme (Mazzucato *et al*., 2023). This is particularly concerning because crop water requirements will outstrip supply by *c*. 40% by 2030 (Lee *et al*., 2023). With the demand for food continuing to grow throughout the century (Ritchie & Rodés‐Guirao, 2024), evidence suggests that it will be increasingly difficult to meet global demands. Currently, it is unclear whether rising atmospheric CO_2_ concentration can offset the expected global water deficit of agricultural crops to mitigate yield losses, particularly given the predicted increases in extreme weather events such as heatwaves.

Stomatal optimality (SO) theory posits that stomata function to minimise the amount of water lost per unit of carbon gained (Medlyn *et al*., 2011; Wang *et al*., 2020). When plants display greater stomatal water release (relative to carbon fixed), the slope of *g*_1_ (a parameter of SO theory) is often larger and corresponds with lower iWUE, with *g*_1_ and iWUE inversely related (Medlyn *et al*., 2011, 2017). Under elevated atmospheric CO_2_ concentration, *A* often increases and *g*_sw_ reduces, leading to a decreased *g*_1_ slope and increased iWUE (Gardner *et al*., 2023). On the other hand, during higher temperatures and rising leaf vapour pressure deficit (VPD) (*D*), *g*_1_ is estimated to increase as more water is released from stomata (Lin *et al*., 2015), which implies iWUE will be lower. This is especially true if decoupling of *A* and *g*_sw_ occurs, as is often the case when additional evaporative cooling is required during heatwaves (Marchin *et al*., 2023). At present, it is unclear how wheat SO, *g*_1_, *D* and iWUE will alter during future climates, and an understanding of such responses will be crucial if future resilience is to be maximised.

Opposing wheat leaf surfaces contribute unequally to gas exchange, with the adaxial leaf surface routinely displaying higher *A* and *g*_sw_ under ambient growth conditions (Wall *et al*., 2022). For most other amphistomatous plants (stomata present on both leaf surfaces), this is not the case, with the abaxial leaf surface often providing the larger contribution to gaseous exchanges (Bhagsari *et al*., 1988; Driesen *et al*., 2023; Tulva *et al*., 2025). It is suggested that increases in abaxial leaf surface activity reduce excessive water loss associated with incoming solar radiation, but in wheat, it has been suggested that higher adaxial gas exchanges are required to capture sufficient CO_2_ for *A* that occurs in the underlying mesophyll (Wall *et al*., 2022). We have previously shown that high N‐fertiliser increases the abaxial contribution to overall *g*_sw_ (Caine *et al*., 2024), but it is unclear how rising atmospheric CO_2_ concentration and/or high‐VPD heatwave conditions will also contribute towards how wheat partitions gaseous exchanges between leaf surfaces. Understanding how all these different environmental factors alter leaf water fluxes will be crucial for shedding light on how wheat canopies will use water and cool under future climate extremes.

Under fluctuating light, stomata respond much more slowly (via alterations to *g*_sw_), than the rapid changes that typically occur with *A* (Lawson & Blatt, 2014; Lawson & Vialet‐Chabrand, 2019). Assays have shown that wheat displays a moderate stomatal kinetic response to fluctuating light, but other cereal crops such as rice display faster dynamic responses (McAusland *et al*., 2016). Whilst these findings highlight that there is potential to optimise stomatal kinetics in wheat, it is unclear how wheat stomata will respond to dynamic light changes under future conditions in which atmospheric CO_2_ concentration will be elevated and high‐VPD heatwaves will become more frequent.

Due to the stochastic nature of extreme weather events, we used controlled environmental growth chambers to simulate future growth conditions to investigate the interactive effects of rising atmospheric CO_2_ concentration, high‐VPD heatwaves and drought application on wheat physiology and productivity. Conducting experiments across a gradient of N‐fertiliser treatments, we ask the following questions: (1) Does wheat growing at higher atmospheric CO_2_ concentration maintain enhanced water usage properties during high‐VPD heatwave events? (2) Do wheat stomata on abaxial and adaxial leaf surfaces respond differently to CO_2_ growth concentration and N‐fertiliser application dependent on high‐VPD heatwave application? (3) Do the stomatal dynamics of highly N‐fertilised wheat growing at ambient or elevated CO_2_ concentration become attenuated during high‐VPD heatwaves? And (4) what are the overall impacts on wheat water usage and productivity, when high CO_2_, high‐VPD heatwave treatment, drought and varying N‐fertiliser are considered in combination?

## Materials and Methods

### Plant materials and growth conditions

To study how high‐VPD heatwave application impacted the physiology of wheat (*Triticum aestivum* L.) normally grown under temperate conditions, we chose the elite British wheat variety Mulika (Blackman Agriculture Ltd, Cambridge, UK), which is derived from parents grown in the United Kingdom and continental Europe (Paragon × (Tybalt × Robigus)). Individual plants were grown in 0.8 l pots (IPP, Bytom, Poland) with a 5 : 1 Levington M3 : Perlite compost mix. Experiments were conducted in Convrion BDW40 growth chambers (Winnipeg, Canada), with plants gradually acclimatised to growth conditions over the first 4 weeks of seedling growth. For the first 2 weeks, chambers were set to 15°C day : 10°C night, with a 12 h : 12 h, light : dark cycle with photosynthetically active radiation (PAR) of 1000 μmol m^−2^ s^−1^ provided at canopy level, with lights ramping gradually up and down for 2 h both in the morning and in the evening. This relatively high‐light treatment increased daytime temperatures around the canopy by +4°C, so growth temperatures around plants were *c*. 19°C during the day. Over the next 2 weeks, chamber set temperatures were gradually increased to 23°C during the day and 15°C during the night, with daylength gradually increased from 12 to 14 h. Because of the high‐light intensity, the temperature at canopy level was *c*. 27°C by Week 4. Relative humidity was set to 60% throughout experiments except during heatwaves.

For each CO_2_–VPD growth scenario, we grew and assessed 128 plants (Supporting Information Fig. S1), with deionised water (dH_2_O) applied to pot bases in trays when required. From Day 32 of each growth scenario, plants were divided into four N‐fertiliser treatments (32 plants for each fertiliser treatment: N1–N4), which were then placed into 4× individual trays per N‐fertiliser treatment (4× trays with 8× plants in each tray). Where applicable (N2–N4 plants only), plants were fed with a soluble high‐N fertiliser mix (N : P : K–25 : 15 : 15, with added micronutrients) on a weekly basis for 12 wk (Chempak® High Nitrogen Feed – Formula 2, Thompson and Morgan, Southampton, UK). The 4× trays of N1 control plants received no additional fertiliser, the 4× trays of N2 plants received 2.125 g weekly, the 4× trays of N3 plants received 4.25 g weekly, and the 4× trays of N4 plants received 8.5 g weekly. The total soluble N‐fertiliser for each treatment wk^−1^ was dissolved in 2 l of water and divided equally between the four trays (4 × 8 plants) of the fertiliser grouping (each tray of eight plants received 500 ml of the total fertiliser application). This equated to N2 plants individually receiving *c*. 0.0664 g of fertiliser wk^−1^, N3 plants individually receiving *c*. 0.1328 g and N4 plants receiving *c*. 0.2656 g. Plants that missed N‐fertiliser treatment during drought received additional applications at weekly intervals after the 12 initial applications so that all fertiliser treatment plants received a total of 12 applications. Plants were spaced by *c*. 9–10 cm and were routinely rotated every 1–2 wk within growth chambers. From Day 32, the four trays of each N‐fertiliser treatment were placed immediately adjacently to one another in a 2 × 2 set‐up with *c*. 30 cm between each 32‐plant canopy N treatment. We did not have designated border plants.

Experimental scenarios simulating rising global atmospheric CO_2_ concentration and/or high‐VPD heatwave treatment, across the above‐described range of high‐N fertiliser treatments, are set out in Fig. S1. Plants were grown at 450 ppm (ambient) or 720 ppm (elevated) CO_2_ concentration (2× experiments per CO_2_ treatment), with a 16‐d heatwave treatment (27°C, 60% RH→37°C, 50% RH) supplied at the canopy level (chamber set temperature increased from 23°C→33°C, +4°C for light) to 1× each CO_2_ experiment, starting when flag leaves first emerged. The high‐VPD heatwave for each CO_2_–VPD treatment combination was started on Day 62 for the elevated CO_2_ treatment and Day 64 for the ambient CO_2_ treatment, at a time when flags first became visible. The heatwave treatment increased VPD around the canopy from *c*. 1.43 kPa (ambient) to 3.14 kPa (high).

### Drought treatment

To explore whether high‐CO_2_ concentration could mitigate future high‐VPD heatwave impacts during water deficit, a drought treatment was applied for 12 d (9 d into heatwave treatments), starting from when wheat ears began emerging from canopies. For plants not exposed to heatwave treatments, the 12‐d drought was imposed slightly later due to slower phenological development of emerging ears (Fig. S1). In each of the 4× CO_2_–VPD scenarios, 16 out of 32 plants per N‐fertiliser treatment were droughted by removing all irrigation water from 2× trays of each fertiliser treatment.

### Plant gas exchange measurements

All LI‐6800 gas exchange measurements were undertaken between 10:00 h and 15:00 h on central portions of fully expanded flag leaves, using a LI‐6800 Portable Photosynthesis System with attached Multiphase Flash Fluorometer (6800‐01A) (LI‐COR Biosciences, Lincoln, NE, USA) before any drought treatment. Steady‐state, *A*/*C*_i_ and light–response curves were collected between Days 4 and 8 of heatwave treatments or at an equivalent developmental stage on flag leaves under nonheatwave conditions. For saturating light steady‐state measurements, the leaf chamber conditions were set according to the conditions set within the growth scenario (Fig. S1), except for light intensity, which was set to a saturating light value of 1800 μmol m^−2^ s^−1^ PAR. The flow rate was set to 400 μmol s^−1^, with CO_2_ concentration controlled by CO_2_ reference and temperature by the chamber air temperature (*T*_air_). For each plant, 10 readings from one leaf were taken under steady‐state conditions over a 5‐min period and averaged (*n* = 4–5 plants per fertiliser treatment). For dynamic photosynthesis experiments at saturating light, we assayed 3–4 plants per fertiliser treatment, starting with 5 min of steady‐state measurements, before irradiance was removed for 1 h and then reapplied for an hour. As with steady‐state measurements, conditions apart from light matched the set conditions within the growth chamber for a given scenario.

To produce *A*/*C*_i_ curves to model photosynthetic traits, the same leaf chamber conditions and replication numbers were used as during steady‐state measurements for each CO_2_–VPD growth scenario, except leaf temperature (*T*_leaf_) was set to either 27°C or 37°C rather than *T*_air_. The [CO_2_]_ref_ sequence applied for 450 ppm plants was as follows: 450, 325, 200, 150, 100, 75, 50, 25, 450, 450, 450, 450, 585, 720, 860, 1000, 1250, 1500, 1800. For plants grown at 720 ppm the order of [CO_2_]_ref_ was as follows: 720, 585, 450, 325, 200, 150, 100, 75, 50, 25, 720, 720, 720, 720, 860, 1000, 1250, 1500, 1800 ppm. A match was conducted before each measurement with 3–5 minutes permitted between each [CO_2_]_ref_ treatment for stabilisation. The maximum carboxylation rate of rubisco (*V*_cmax_) and the maximum rate of photosynthetic electron transport (*J*_max_) were modeled using the plantecophys package using the R programming software (Duursma, 2015; R Core Team, 2021).

Leaf *g*_sw_ and *E* were collected from fully expanded flag leaves between 10:00 h and 14:00 h using a LI‐COR LI‐600 porometer set to a flow rate of 150 μmol s^−1^ on Day 5 of heatwave treatments, or 5 d after flag leaves began emerging for nonheatwave plants. For porometry data collected before drought treatment (before ear emergence), a total of 32 plants per N treatment were measured per CO_2_–VPD growth scenario, with this number reduced to 16 each for droughted and well‐watered plants during the drought. For the drought experiment, measurements were undertaken beginning from when ears emerged in each treatment (Day 0), with subsequent measurements collected every 3 d until Day 12 of the drought. For all plants across all N‐fertiliser treatments at all time points, both leaf surfaces were measured individually using the porometer, with the data presented showing either individual leaf surface results separately or the combination of both leaf surfaces added together. To compute the % abaxial contribution to leaf *E* or *g*_sw_, abaxial and adaxial surface values were first added together, and then the abaxial surface value was divided by the value of both sides combined. This proportion was then multiplied by 100× to obtain the % contribution of the abaxial leaf surface.

### Analysis of leaf stomatal traits

For stomatal size (SS) and stomatal density (SD) quantification (*n* = 8), impressions were collected from the central portions of mature flag leaves of well‐watered plants on Day 16 of the heatwave (on Day 7 of the drought for nonheatwave plants). ImpressPLUS Wash Medium Body Fast Set dental resin (Perfection Plus Ltd, Dublin, Ireland) was used to acquire impressions, which were applied to both the abaxial and adaxial leaf surfaces simultaneously. The resin was allowed to set for *c*. 30 min in growth chambers before removal. Clear 60 s Super Shine Nail Polish (Rimmel, London, UK) was applied to flag leaf dental resin impressions, by applying two coats of nail polish, allowing 30 min per application for drying. Nail polish impressions were then peeled off the resin and transferred to glass slides, with a glass cover slip affixed atop to secure the sample before imaging.

Stomatal imaging was conducted using a 10× objective lens on a Brunel N‐300 M microscope (Brunel Microscopes Ltd, Chippenham, UK), equipped with a Moticam 5 (Motic, Kowloon Bay, Hong Kong), set to a resolution of 2592 × 1944 pixels. A graticule image was captured at the same resolution to enable size and area measurements from images. For each biological replicate and each leaf side, two fields of view (FOV) were imaged using the fiji imagej2 imaging software (Schindelin *et al*., 2012). The same equivalent areas of all leaves were assessed, focussing on the region inside of the second major vertical vein away from the leaf edge (1× FOV from each leaf edge, totalling two FOVs for each leaf surface). The areas imaged had two minor vertical veins located centrally with the image, without the second major vertical vein being in the FOV. To calculate SS, the distance from the top to the bottom of five randomly selected guard cells (GCs) was measured and these values were averaged to give an average value per FOV. To calculate SD in the form of stomata mm^−2^, the number of stomata counted was divided by the total area counted (total area = 1.2049 mm^2^). To get a biological replicate value for each leaf surface for SS and SD, both individual FOV values of the respective traits were averaged.

### Thermal imaging

Thermal images of plants were captured between 10:00 h and 14:00 h using a FLIR T540 thermal imaging camera (Wilsonville, OR, USA), at equivalent time points to when porometry measurements were being undertaken across different CO_2_–VPD treatments. This was the case both for predrought and drought porometry measurements. The emissivity was set to 0.95 for all measurements. One leaf from each plant per fertiliser treatment (*n* = 32) for each CO_2_–VPD growth scenario was measured and its surface temperature subtracted from the temperature of the green plastic hemispherical dry reference surface placed within the growth chamber to calculate ∆ leaf temperatures relative to the reference surface. Image analysis was conducted using FLIR Researcher IR MAX (www.flir.co.uk).

### Leaf biochemical and structural measurements

For analysis of leaf Chl content, 2 × 6‐mm‐diameter leaf discs were collected at Day 8 of heatwaves or an equivalent phenological time point after flag leaves began emerging in nonheatwave treatments (before ear emergence). Samples were weighed and placed into 5 ml of N,N‐dimethylformamide ≥ 99.8% (Thermo Scientific, Oxford, UK) and stored at 4°C for 7 d to enable Chl extraction. A total of 32 plants were measured per N‐fertiliser treatment for each of the 4× growth scenarios. Chl absorbance was measured at the following wavelengths: 663.8, 646.8 and 480 nm (Wellburn, 1994), with a Shimadzu UV2600i spectrophotometer (Kyoto, Japan). Chl content was normalised by mass by dividing a dry weight leaf sample by a fresh weight leaf sample and then multiplying this value by the weight of the collected leaf discs.

### Hyperspectral imaging and data analysis

A PSR+ spectroradiometer (Spectral Evolution, Haverhill, MA, USA) was used to collect leaf‐level hyperspectral reflectance data (350–2500 nm), utilising an attached Spectral Evolution leaf clip assembly. Measurements were taken on the central portions of adaxial leaf surfaces between 08:00 h and 10:00 h. For readings collected before drought, a total of 32 plants per N‐fertiliser treatment were measured within each CO_2_–VPD treatment experiment on the same day leaf Chl samples were collected. During drought, between 8 and 16 plants were assessed per N‐fertiliser treatment. Target and reference panel measurements from a radiometrically calibrated 99% Spectralon panel were sampled sequentially. The spectrolab package was used to import hyperspectral data in R (Meireles *et al*., 2017). We used the MERIS terrestrial Chl index (MTCI) ((*R*_754_ *–R*_709_)/(*R*_709_–*R*_681_)) as an approximation for Chl content as we have previously found this to be a robust index for quantifying Chl in wheat (Caine *et al*., 2024).

### Stomatal optimisation modelling

The optimal stomatal conductance under different CO_2_ and VPD growth treatments was calculated following the theory and approach of Medlyn *et al*. (2011) and with alterations based on Medlyn *et al*. (2017):

(Eqn 1) $$g_{s}\approx 1.6\left(1+\frac{g_{1}}{\sqrt{D}}\;\right)A_{\mathrm{net}}/C_{a}$$

where *g*_s_ is the stomatal conductance, *D* is the leaf–air vapour pressure difference (kPa), *A*_net_ is measured photosynthesis, and *C*_a_ is the CO_2_ concentration outside the leaf. To estimate *g*_1_, we used:

(Eqn 2) $$g_{1}=\frac{\left(\frac{C_{i}}{C_{a}}\sqrt{D}\right)}{\left(1-\frac{C_{i}}{C_{a}}\right)}$$

where *C*_i_ refers to the CO_2_ concentration inside the leaf. In addition to the √*D* (*D* ^0.5^), we also assessed *D* ^0.2^ and *D* ^0.8^ within Eqn (2) to explore whether SO modelling accuracy could be enhanced. For each N and CO_2_–VPD treatment, 4–5 plants were assessed and modelled.

### Statistical analysis

Statistical analysis was conducted using the R programming software (R Team, 2021). To test for data normality before conducting ANOVA and/or generalised linear model (GLM) application, the residuals of datasets were analysed using the shapiro.test function from the stats package, with variance tested via the leveneTest function from the car package (Fox & Weisberg, 2019). Where data was normally distributed and variance equal, one‐way, two‐way or three‐way ANOVA tests were undertaken using the aov function from the stats package, and then, the Tukey's HSD function was used for *post hoc* testing (R Team, 2021). When the variance was not equal, a Welch test was undertaken instead of a two‐way ANOVA using the oneway.test function, and then, a *post hoc* test was undertaken using the gamesHowellTest function from the pmcmrplus package (Pohlert, 2024).

When non‐normal data were detected and/or variance was unequal, three‐way GLMs were undertaken utilising the stats package, with the appropriate distribution selected for the GLM that best fitted the data (R Team, 2021). GLMs were then assessed using the anova function from the stats package to produce an analysis of deviance table to assess whether CO_2_, VPD and/or fertiliser treatments were significant. For multiple comparisons, the emmeans function from the emmeans package was used to compute the estimated marginal means (EMM) from GLMs, and the compact letter display of pairwise comparisons (cld) function was applied with the Sidak adjustment to determine statistical differences between different samples within each N treatment or CO_2_–VPD treatment (Hothorn *et al*., 2008; Lenth, 2025). For significant three‐way ANOVAs, a linear model was first produced, which was used to compute EMMs before the cld function was applied with the Sidak adjustment.

To assess leaf surface percentage contributions, data were arc‐sine‐transformed using the following formula from baseR (R Team, 2021), where *x* denotes the abaxial proportion to total gas exchange:

(Eqn 3) $$\text{asin}\left(\text{sqrt}\left(x\right)\right)$$

As some negative values for abaxial leaf surface *g*_sw_ were present for nonheatwave high CO_2_ plants, these were removed from the analysis, as such values could not be transformed. For specific statistical tests and replicate number per treatment, see individual figure legends.

## Results

### During high‐VPD heatwaves, wheat releases more water from stomata irrespective of CO_2_ growth concentration

**Fig. 1:**
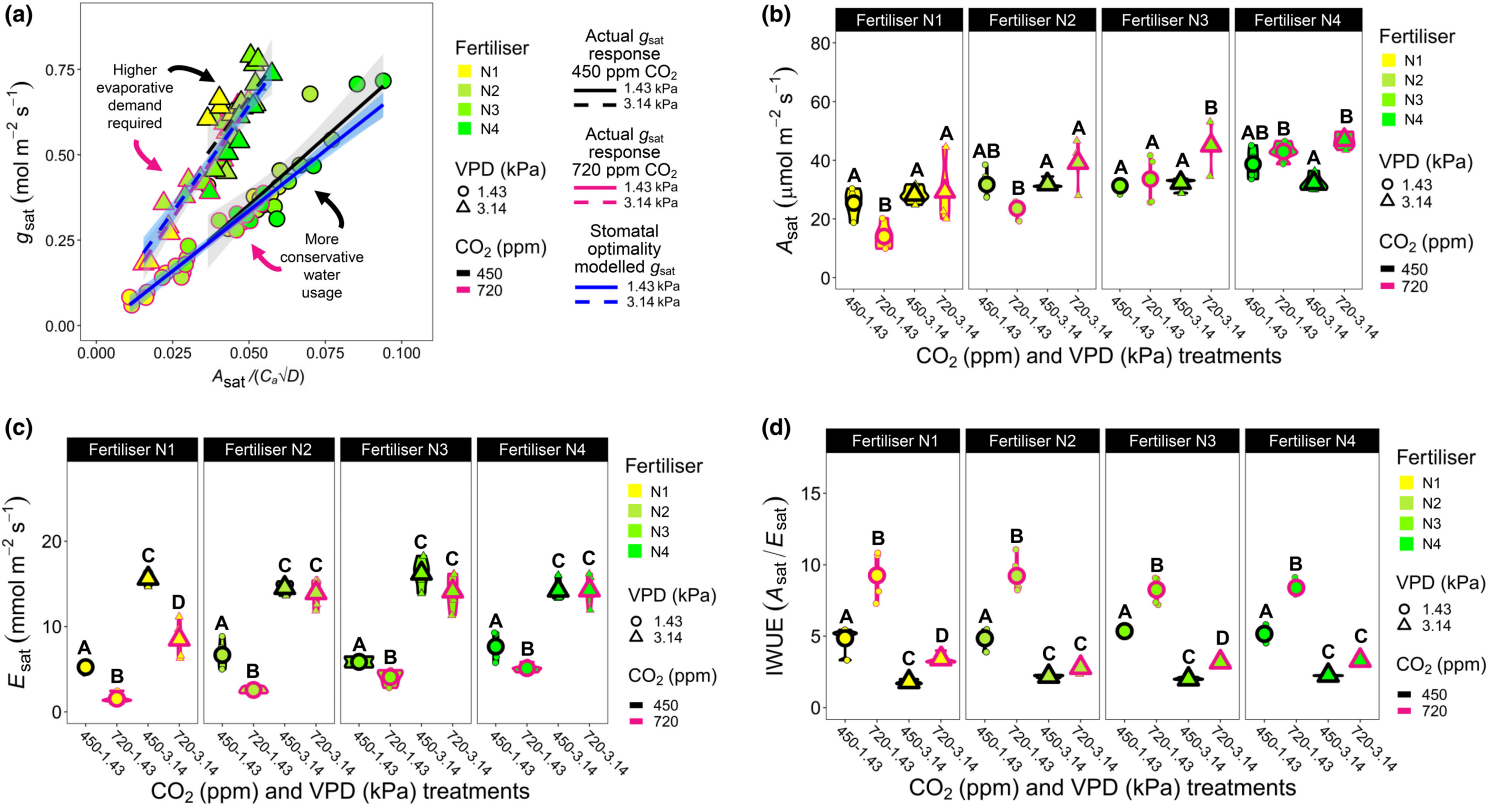
Stomatal optimality (SO) modelling of wheat carbon and water fluxes under current and future heatwave conditions. (a) Optimality modelled and actual gas exchange responses, under differing nitrogen (N) fertiliser, CO_2_ concentration and vapour pressure deficit (VPD) conditions. The *y*‐axis denotes light‐saturated stomatal conductance (*g*_sat_). The *x*‐axis represents a measure of photosynthetic output based on the saturating light photosynthesis (*A*_sat_), divided by the ambient CO_2_ concentration (*C*_a_) multiplied by the square root of the leaf to air VPD (√*D*). Black (450 ppm) and pink (720 ppm) solid and dashed lines and symbols represent regression analyses alongside measured values. The solid blue line represents SO modelled *g*_sat_ data at 1.43 kPa VPD (nonheatwave) and dashed blue lines represent SO modelled data *g*_sat_ at 3.14 kPa (heatwave). The *D* parameter within the *g*_1_ formula of the SO modelled results presented (solid and dashed blue lines) is set to *D* ^0.8^ rather than the √*D*, with additional parametrisations of *g*_1_ *D*, including √*D* being presented in Supporting Information Fig. S2. (b) *A*_sat_, (c) saturating light transpiration (*E*_sat_) and (d) instantaneous water‐use efficiency (IWUE; *A*_sat_/*E*_sat_) across different N‐fertiliser, CO_2_ concentration and VPD treatments. For all panels, *n* = 4–5. Larger symbols equal sample means except in a where they represent individual values. For (b, c), three‐way ANOVAs were performed to test for overall significance. In (d), a three‐way generalised linearised model (GLM) was employed. In all cases, estimated marginal means were computed to probe for differences within N‐fertiliser groupings, with the cld function utilised with Sidak adjustment applied to detect significance between growth treatments. Within individual graphs, different letters indicate significant differences of *P <* 0.05. See Tables S1–S3, for statistical information relating to the significance of treatments and potential interactions for three‐way ANOVA and GLM analyses.

To investigate how wheat adjusts stomatal water and carbon fluxes during high‐VPD heatwaves (3.14 kPa compared to 1.43 kPa control) under different CO_2_ concentrations (450 or 720 ppm), we modelled gaseous exchanges across a range of N‐fertiliser treatments using SO theory (Medlyn *et al*., 2011, 2017; Lu *et al*., 2020; Gardner *et al*., 2023) (Fig. 1a). Gas exchange data including light saturated *A* (*A*_sat_) and *E* (*E*_sat_) are also presented, along with instantaneous water‐use efficiency (IWUE; *A*_sat_/E_sat_) results for four N‐fertiliser treatments, across four CO_2_–VPD treatment combinations (Figs 1b–d, S2, S3; Tables S1–S7).

Fig. 1(a) demonstrates that wheat leaves exhibited increased water release during high‐VPD heatwave conditions irrespective of CO_2_ concentration (Welch's *t*‐test, test statistic = 0.35, *P* = 0.73). As we found the standard SO model to provide only a reasonable fit of our measured data, we attempted different parametrisations of *D* within *g*_1_ estimation and found *D* ^0.8^ (Fig. 1a) to be more accurate than √*D* (*D* ^0.5^) for modelling *g*_sat_ (Fig. S2). When we compared *A*_sat_ between growth treatments, we found under nonheatwave conditions that high‐CO_2_ N1 plants had significantly lower *A*_sat_ than ambient CO_2_ equivalent plants, whereas this was not the case for N2–N4 plants, which were not significantly different between CO_2_ treatments (three‐way ANOVA, EMMs assessed with the cld function applied with Sidak adjustment; Fig. 1b; Table S1). The different *A*_sat_ responses to N between CO_2_ treatments at low N highlight the importance of sufficient N‐fertiliser for achieving increased *A*_sat_ at elevated CO_2_ concentration. High‐VPD heatwave treatment enhanced *A*_sat_ for wheat receiving higher N‐fertiliser quantities, but only for high CO_2_ grown plants, with N3 and N4 high CO_2_ heatwave plants having significantly higher *A*_sat_ than ambient CO_2_ equivalent plants (three‐way ANOVA, EMMs assessed with the cld function utilising the Sidak adjustment, N3 = *P* < 0.001, N4 = *P <* 0.0001; Figs 1b, S3a; Table S1).

Assessment of *E*_sat_ revealed nonheatwave, high‐CO_2_ plants to have lower *E*_sat_ than ambient CO_2_ equivalent plants across all 4 N‐fertiliser treatments (Figs 1c, S3b). During high‐VPD heatwaves, only N1 high CO_2_ plants had significantly reduced *E*_sat_ compared with ambient CO_2_ plants (three‐way ANOVA, EMMs assessed with the cld function utilising the Sidak adjustment, *P* < 0.01; Table S2), with no significant differences detectable between N2 and N4 N‐fertiliser plants when ambient and high‐CO_2_ high‐VPD heatwave plants were compared (Fig. 1c). Overall, we found similar saturating light stomatal conductance (*g*_sat_) responses to *E*_sat_ responses, although *E*_sat_ values more clearly highlighted water flux differences between VPD treatments (Figs 1c, S3c). This move towards greater water release during heatwaves was reflected in lower IWUE (based on *E*_sat_) and iWUE (based on *g*_sat_) values, with particularly large decreases of IWUE for plants grown at high CO_2_ (Figs 1d, S3d). The increased water release during high‐VPD heatwaves was also linked with significantly higher leaf VPD values, which also often corresponded to higher *C*_i_ : *C*_a_ ratios (Fig. S3e,f).

**Fig. 2:**
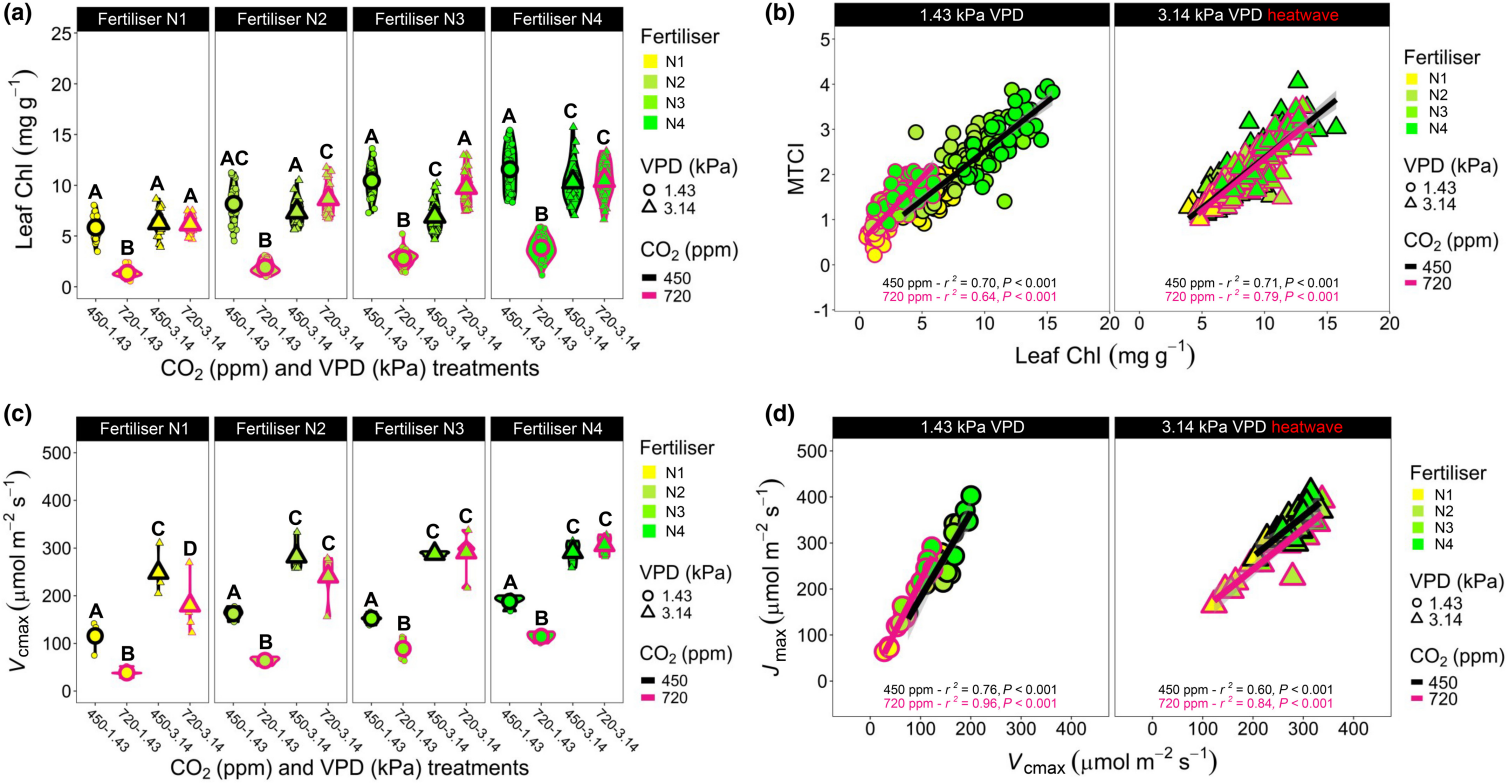
Variation in wheat leaf Chl content and photosynthetic productivity based on different nitrogen (N) fertiliser, CO_2_ concentration and/or heatwave application. (a) Leaf Chl content by mass. (b) Regression analyses of leaf Chl and MERIS terrestrial Chl index from plants grown under the four different environmental treatments. (c) The maximum rate of rubisco carboxylation (*V*_cmax_) and (d) regression analyses between *V*_cmax_ and the maximum rate of electron transport (*J*_max_). For (a, b), *n* = 31–32; for (c, d), *n* = 4–5. In (a, c, d), larger symbols equal sample means, whereas in (b), large symbols are independent replicates. For both (a, c), three‐way generalised linear models (GLMs) were employed with estimated marginal means computed to probe for differences within N‐fertiliser groupings, with the cld function and Sidak adjustment applied to detect significance between growth treatments. Within individual graphs, different letters indicate significant differences of *P ≤* 0.05. See Supporting Information Tables S8 and S9 for statistical information relating to the significance of treatments and potential interactions for GLM analyses.

To assess the variation in underlying physiological traits that impacted heatwave gaseous exchanges, we collected leaf Chl and hyperspectral data, and measured physiological parameters associated with photosynthetic performance (Figs 2, S4; Tables S8–S10). Nonheatwave, high‐CO_2_‐grown plants consistently yielded the least Chl by mass, across all N‐fertiliser applications and the addition of a high‐VPD heatwave treatment to high‐CO_2_ growth conditions led to a reversal of this trait response (Figs 2a, S4a). Hyperspectral data acquisition (Dataset S1) confirmed the observed relationship between N‐fertiliser application and Chl across different growth scenarios, with the MTCI vegetation index and Chl by unit mass displaying strong correlations across CO_2_–VPD treatment combinations (Fig. 2b).

Calculation of *V*_cmax_ at the respective growth temperatures revealed high‐VPD heatwave treatment always increased *V*_cmax_ values for both CO_2_ treatments within N‐fertiliser treatments, whereas this was not always the case for the maximum rate of photosynthetic electron transport (*J*_max_), as nonheatwave and heatwave values remained similar for highly fertilised N3 and N4 plants grown and measured at ambient CO_2_ concentration (Figs 2c,d, S4b). These larger increases in *V*_cmax_ during heatwaves led to clear differences in the slopes of nonheatwave and heatwave‐treated wheat plants when *V*_cmax_ and *J*_max_ were regressed (Fig. 2d). *A*/*c*_i_ curve observations revealed high CO_2_ plants were often RuBP‐limited under elevated CO_2_ growth concentration (N2–N4 plants), whereas ambient CO_2_ plants were rubisco‐limited (Fig. S5). Supply function comparisons between ambient and high‐CO_2_ treatments revealed that stomatal limitations were the same between highly N‐fertilised plants experiencing high‐VPD heatwave conditions, but this was not the case under lower N‐fertiliser treatments and/or when plants were assessed under ambient VPD, in which higher CO_2_ treatment did appear to restrict gaseous diffusion (Fig. S5).

### Enhanced evaporative cooling during high‐VPD heatwaves is primarily achieved via increased water loss from stomata on the abaxial leaf surface

**Fig. 3:**
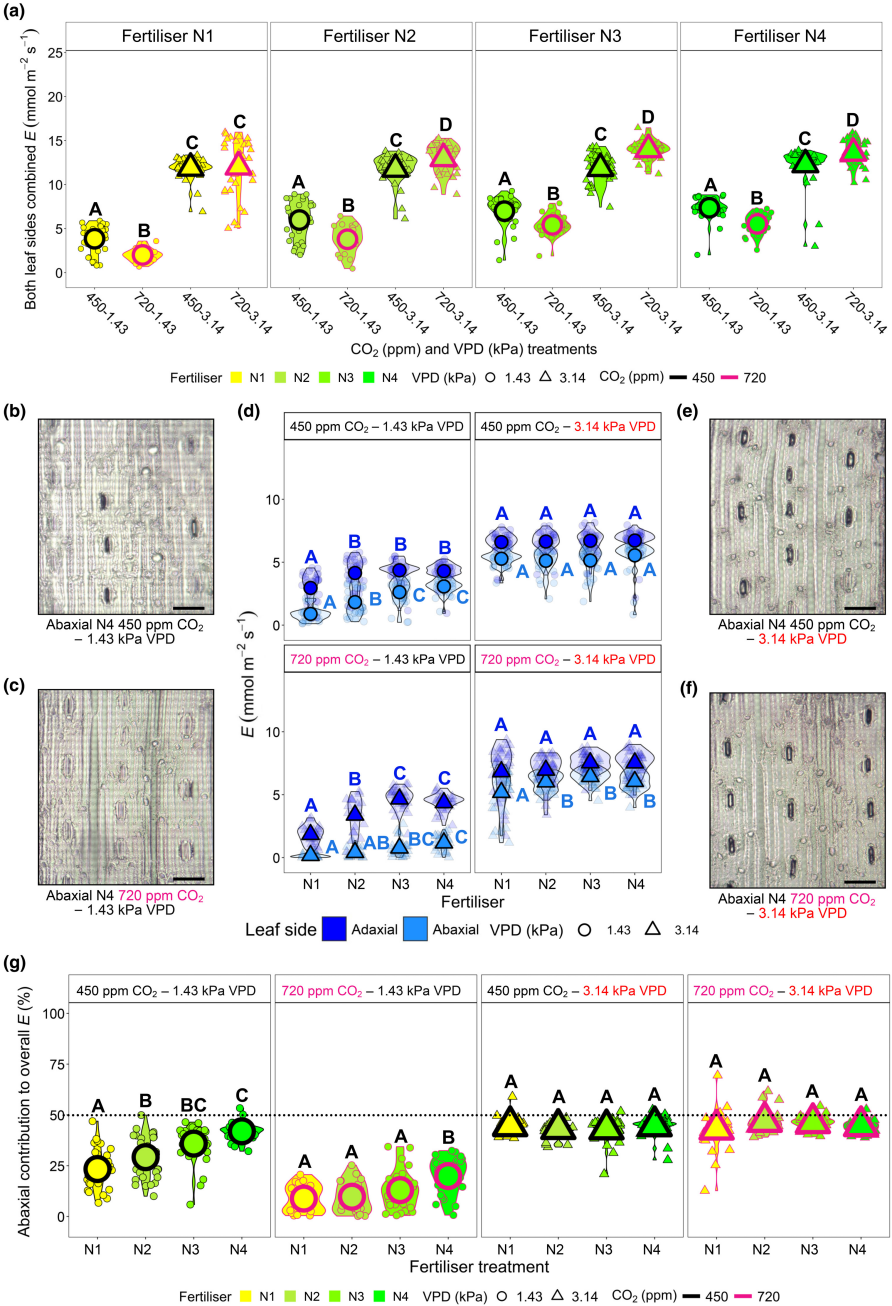
Abaxial and adaxial leaf surface contributions to wheat water fluxes under current and future heatwave scenarios. (a) Porometer measured total leaf transpiration (*E*) across different vapour pressure deficit (VPD; 1.43 or 3.14 kPa), CO_2_ concentration (450 or 720 ppm) and nitrogen (N) fertiliser treatments, measuring both leaf surfaces 5 d into high‐VPD heatwaves (or 5 d after flag leaves emerged during nonheatwaves). (b–f) Representative abaxial leaf surface images of the 4× CO_2_–VPD treatment scenarios with quantification of individual *E* measurements (d) on either surface. (g) Abaxial leaf percentage contribution to total *E* under different CO_2_ and VPD treatments. Bars: (b, c, e and f), 100 µm.  For (a, d), *n* = 32; for (g), *n* = 20–32. Large symbols within graphs equate to sample means. For all statistical testing, three‐way generalised linear models (GLMs) were undertaken with estimated marginal means computed to probe differences within N‐fertiliser groupings for (a), or within treatment categories (CO_2_–VPD) in (d, g), with the cld function and Sidak adjustment applied to detect significance between treatments. Within individual graphs, different letters indicate significant differences of *P <* 0.05. See Supporting Information Tables S11–S14, for statistical information relating to the significance of treatments and potential interactions for three‐way GLM analyses.

The increase in water demand during high‐VPD heatwaves is further explored for both leaf surfaces in Fig. 3.

Previously, and in data presented here, we have shown that higher N‐fertiliser application enhances leaf *E* and *g*_sw_ on both the abaxial and adaxial leaf surfaces during ambient growing conditions (Caine *et al*., 2024) (Figs 3, S6, S7; Tables S11–S18). Under ambient VPD (nonheatwave) growing conditions, total leaf *E* (abaxial and adaxial leaf surfaces combined) was consistently lower for high‐CO_2_ plants than for ambient CO_2_ plants across all N‐fertiliser treatments (Figs 3a, S7a). Strikingly, high‐VPD heatwave exposure resulted in an opposite response in three out of four N‐fertiliser treatments, with high N‐fertilised, high CO_2_ plants having higher leaf *E* than ambient CO_2_ heatwave plants (Fig. 3a). Gradual increases in *E* in response to increasing N‐fertiliser were not as clear during high‐VPD heatwaves, with stomatal opening and *E* insensitive to increasing N‐application during ambient CO_2_ concentration heatwave treatment (Figs 3b–g, S7a,b). Leaf *E* increases during heatwaves were particularly evident on the abaxial leaf surface, in which *E* contribution to total gas exchange rose from a low of 9% during nonheatwave conditions, to 43–47% during heatwaves (Figs 3g, S7c). Whilst not indicative of overall water flux differences, abaxial *g*_sw_ responses often followed similar trends to *E* (Fig. S7d–h).

**Fig. 4:**
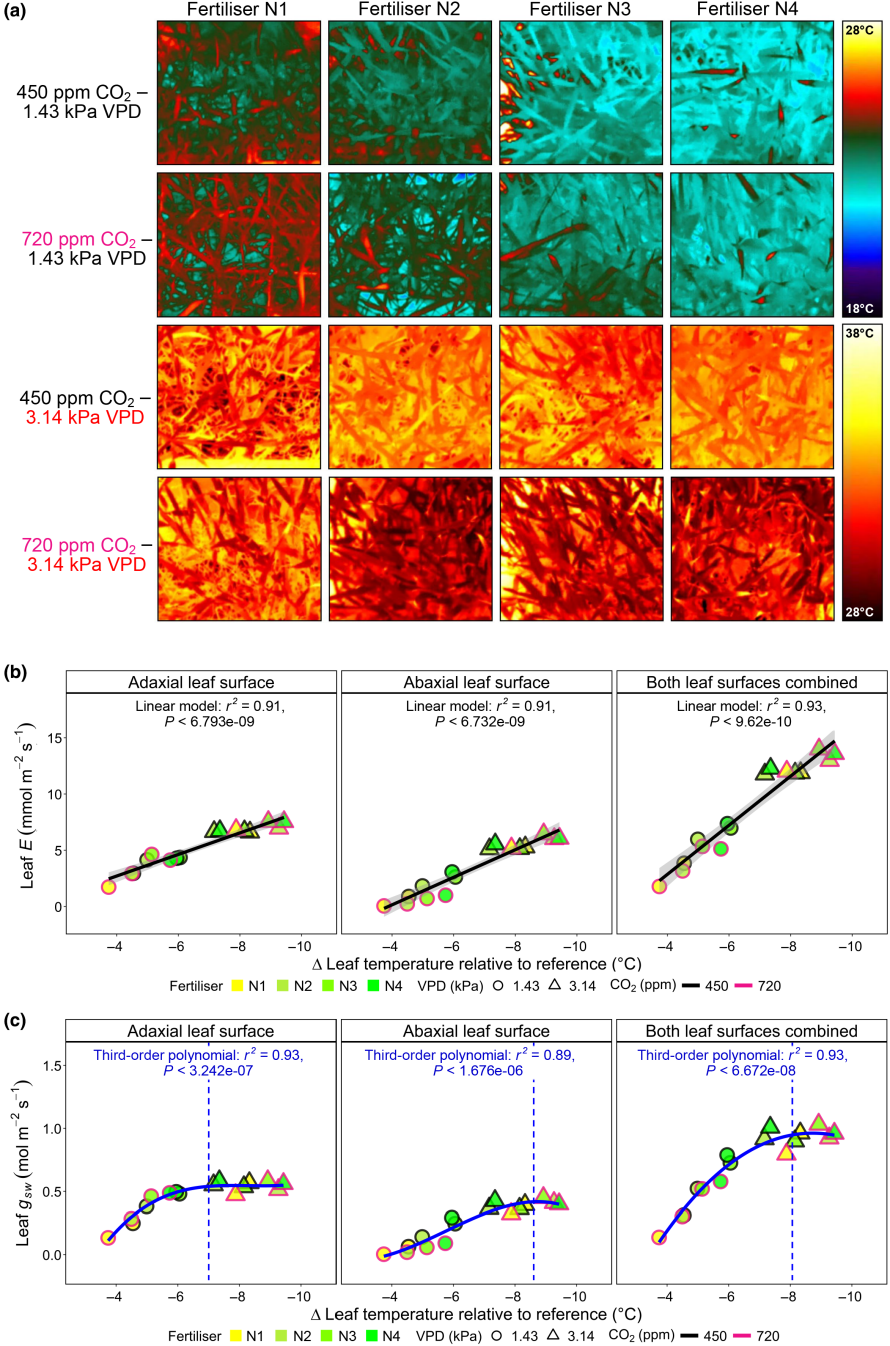
Thermal profiling of wheat canopy evaporative cooling during heatwave treatment. (a) Thermal images of wheat growing under different CO_2_ (450 or 720 ppm), vapour pressure deficit (1.43 or 3.14 kPa) and nitrogen (N) fertiliser (N1–N4) treatments. Regression comparison of (b) average leaf transpiration (*E*) or (c) stomatal conductance to water vapour (*g*_sw_) with average Δ leaf temperature, considering adaxial, abaxial or both leaf surfaces together. For analyses in (b), linear regressions are applied, and in (c), third‐order polynomial regressions are undertaken. To calculate the Δ leaf temperature, the actual temperature of a leaf was subtracted from the temperature of a hemispherical reference placed within the corresponding growth chamber. Each large symbol in individual regressions represents a mean of a treatment, with *n* = 32 for each treatment.

Thermal imaging of wheat canopies (Fig. 4a–c) supports leaf‐level porometry measurements of *E* presented in Fig. 3. This was particularly evident for heatwave treatment differences, with high‐CO_2_ plants noticeably cooler than ambient CO_2_ equivalents across N2, N3 and N4 fertiliser treatments. (Figs 4a, S8a,b; Table S19). This increase in water fluxes was also often detectable in the whole‐plant water usage of N‐fertilised plants during the first 5 d of the heatwave (Fig. S8c; Table S20).

When relationships between adaxial and/or abaxial leaf *E* and ∆ leaf temperature were assessed across treatments, a strong positive correlation was detected for individual leaf sides when regressed against ∆ leaf temperature (both *R* ^2^ = 0.91, *P <* 0.001), and an even stronger relationship was detectable when both leaf side *E* values were combined (*R* ^2^ = 0.93, *P <* 0.001; Fig. 4b). Corresponding *g*_sw_ measurements revealed that maximum values of adaxial leaf *g*_sw_ occurred when ∆ leaf temperature reached *c*. −7°C, whereas for the abaxial surface, maximum *g*_sw_ was closer to −9°C (Fig. 4c). These responses highlight the additive effect of abaxial *g*_sw_ on plant cooling during high‐VPD heatwaves.

### High VPD slows stomatal movements irrespective of CO_2_ concentration

**Fig. 5:**
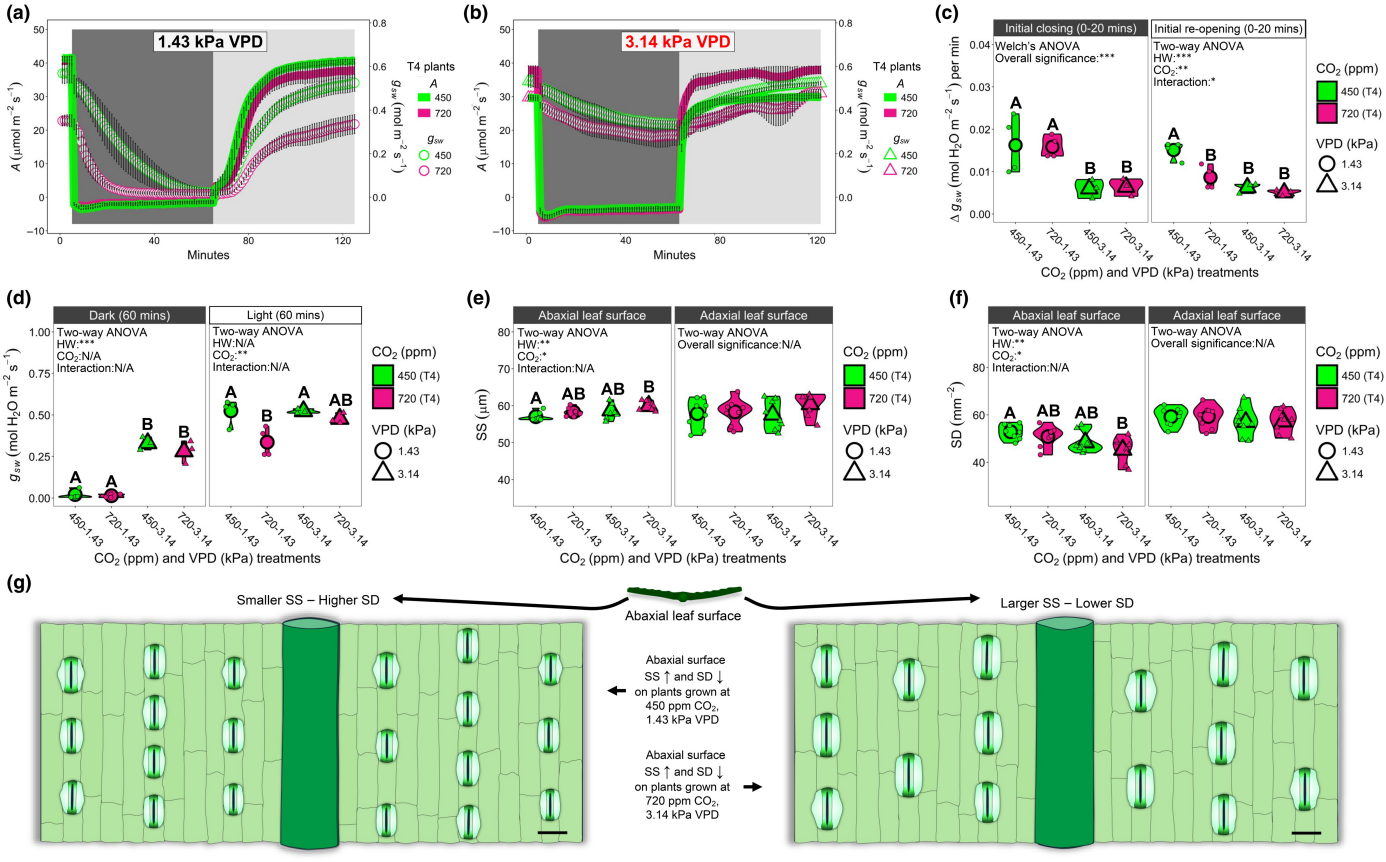
Light‐saturated light‐shift experiments on wheat measured under corresponding growth scenario experiments. (a, b) Photosynthesis (*A*) and stomatal conductance (*g*_sw_) measurements of (a) Nonheatwave (vapour pressure deficit (VPD): 1.43 kPa) and (b) high‐VPD heatwave (VPD: 3.14 kPa) N4 treated plants under ambient (450 ppm) and elevated (720 ppm) CO_2_. (c) Initial *g*_sw_ rate changes min^−1^ during closure and re‐opening period (20‐min durations for each) for (a, b). (d) Measurements of *g*_sw_ at the end of the dark period (65 min) and light re‐application (125 min). Abaxial and adaxial (e) stomatal size (SS) and (f) stomatal density (SD) measurements. (g) SS and SD alterations in response to combined CO_2_ and VPD treatments. For (a–d) *n* = 3–4, for (e, f) *n* = 8. Large symbols equal sample means. For two‐way ANOVAs, Tukey's *post hoc* tests were performed. For Welch's ANOVAs, Games–Howell tests were undertaken. Different letters indicate significant differences of *P <* 0.05. *, *P* < 0.05; **, *P <* 0.01; ***, *P* < 0.001.

Enhancing stomatal responsiveness during changing environmental conditions is a key focus for boosting crop iWUE and *A* (McAusland *et al*., 2016). Light‐shift experiments (saturating light steady‐state: 5 mins → dark: 1 h → saturating light: 1 h) were undertaken on highly N‐fertilised, N4 wheat plants under the four different CO_2_–VPD scenarios (Figs 5, S1, S9).

For nonheatwave, high‐CO_2_ plants, *A* was initially similar to ambient CO_2_ plants, whereas *g*_sw_ was significantly lower (two‐way ANOVA, *P* < 0.01; Figs 5a, S9a,b). For the high‐VPD heatwave treatment, high‐CO_2_ plants started with significantly higher *A* than ambient CO_2_ plants (two‐way ANOVA, *P* < 0.001), whereas *g*_sw_ values were not significantly different (Figs 5b, S9a,b). When the dark treatment was applied during nonheatwave conditions, ambient CO_2_ plants took much longer for stomata to close than high‐CO_2_‐treated plants (*c*. 60 min for ambient CO_2_, relative to *c*. 20 min for high CO_2_) (Fig. 5a). This seemed to occur as *g*_sw_ started considerably higher for ambient CO_2_ plants (meaning stomata were more open to start with), but overall, the initial rate of stomatal closure min^−1^ during the first 20 min of dark was not significantly different between CO_2_ treatments (Fig. 5a,c). Heatwaves considerably hampered stomatal closing for both CO_2_ treatments, with *g*_sw_ values only reducing by *c*. 37–38%, and no rate change differences were detectable between ambient and high‐CO_2_ plants (Fig. 5b–d).

Upon re‐application of saturating light, ambient CO_2_, nonheatwave plants had a larger *g*_sw_ rate change min^−1^ than high‐CO_2_ grown plants (Fig. 5a,c). Heatwave conditions produced smaller *g*_sw_ rate changes min^−1^ than occurred for nonheatwave plants, with no detectable difference between ambient and high‐CO_2_ plants (Fig. 5b,c). After 60 min of saturating light, ambient CO_2_ plants again had significantly higher *g*_sw_ than high‐CO_2_ plants (under nonheatwave conditions), but this was not the case for heatwave‐treated plants, in which *g*_sw_ values between CO_2_ treatments were similar (Fig. 5d). We also assessed the impact of saturating light on leaf temperatures (by assessing *T*_leaf_ values) and found that light application added *c*. 1–1.6°C of temperature to leaf samples when leaves were being irradiated (Fig. S10). Assessing *T*_leaf_ also highlighted that heatwave plants always maintained temperatures below the set air temperature, whereas this was not always the case for plants that were grown and assessed under ambient VPD conditions in which *T*_leaf_ and *T*_air_ remained similar. To further evaluate stomatal responsiveness to higher VPD, we also re‐assessed *E* and *g*_sw_ values from *A*/*C*_i_ curves of N4 plants and found that heatwave treatment led to ‘CO_2_ blindness’ at higher CO_2_ reference concentrations irrespective of CO_2_ growth concentration (Fig. S11). Normally, stomata should begin to close as higher CO_2_ reference concentrations are supplied to a leaf during an *A*/*C*_i_ curve (as we detected during our ambient temperature treatment *A*/*C*_i_ curves), leading to reductions in *E* and *g*_sw_. This was not the case however for heatwave plants at either CO_2_ concentration; instead, *E* and *g*_sw_ remained constant as CO_2_ reference concentrations were increased (Fig. S11).

SS and SD assessment on both leaf surfaces revealed the abaxial leaf surface to be more responsive to different growth conditions, whereas the adaxial surface did not show such plasticity in response to the environment (Fig. 5e–g). Overall, both CO_2_ concentration and VPD treatment affected abaxial SS, with higher CO_2_ and higher VPD increasing SS (two‐way ANOVA, CO_2_ = *P* < 0.05, HW = *P* < 0.01; Fig. 5e). Increased VPD also significantly reduced abaxial SD (two‐way ANOVA, *P* < 0.01), and the combination of both high CO_2_ and VPD led to significant reductions in SD compared to plants grown under ambient CO_2_ and VPD (two‐way ANOVA, Tukey's HSD, *P* < 0.01; Fig. 5f).

### High‐VPD heatwave conditions increases vulnerability to drought and limits yield

**Fig. 6:**
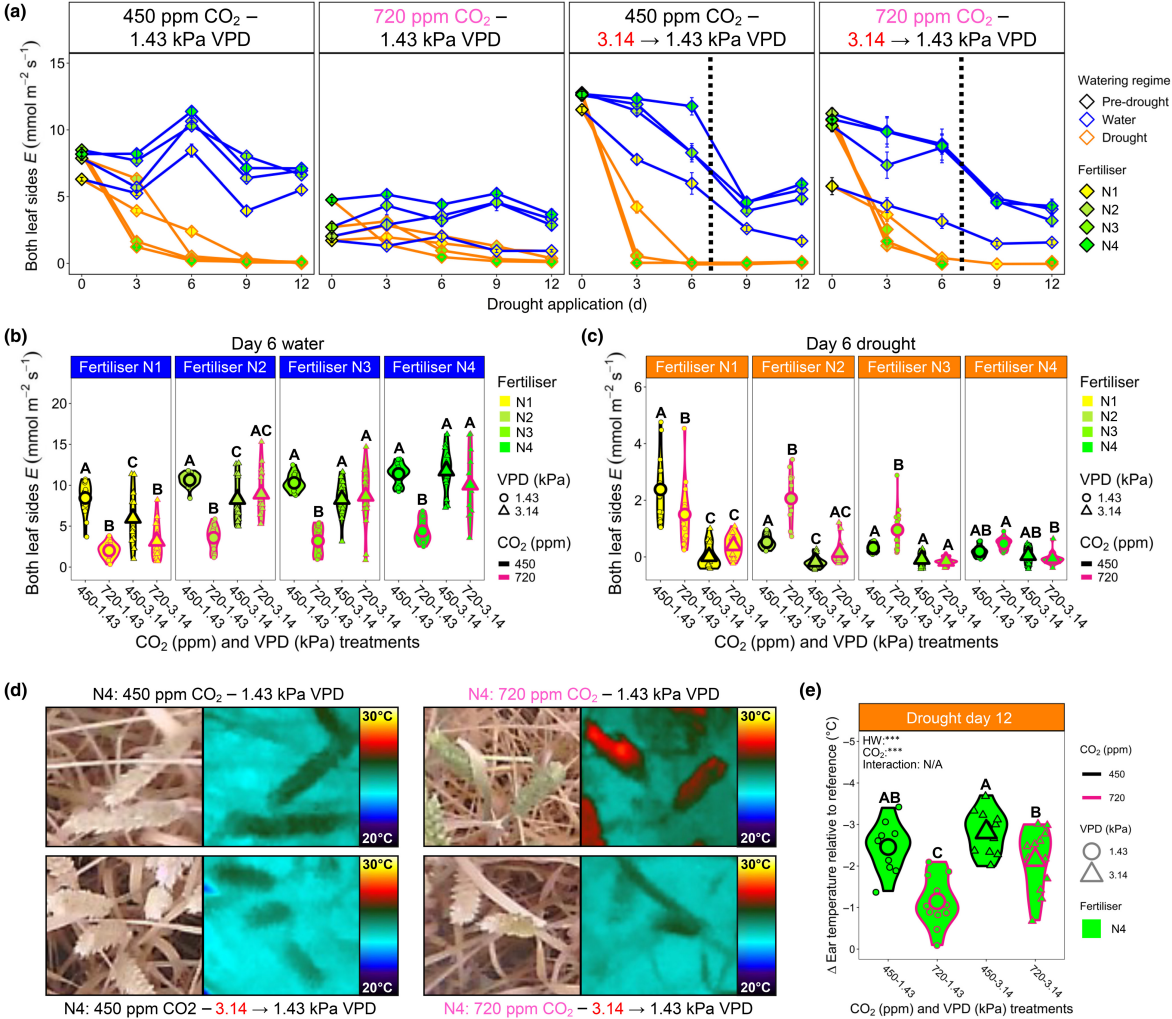
Wheat drought resilience under current and future high vapour pressure deficit (VPD) heatwave conditions. (a) Combined leaf surface transpiration (*E*) during drought across the four different CO_2_ and VPD combinations. (b, c) Day 6 quantification of *E* values for (b) continually watered plants and (c) droughted plants. (d) Thermal and digital images of wheat ears at Day 12 of the drought. (e) Droughted wheat ear Δ ear temperature relative to the reference surface at Day 12. For (a–c): *n* = 16, for (e) *n* = 12. Large symbols equal sample means. For both (b, c), three‐way generalised linear models (GLMs) were employed, with estimated marginal means computed to probe for differences within nitrogen (N) fertiliser groupings, with the cld function and Sidak adjustment applied to detect significance between growth treatments. In (e), a two‐way ANOVA was conducted followed by a Tukey's *post hoc* test. Within individual graphs, different letters indicate significant differences of *P <* 0.05. *, *P* < 0.05; **, *P <* 0.01; ***, *P* < 0.001. See Supporting Information Tables S21 and S22, for statistical information relating to the significance of treatments and potential interactions for three‐way GLM analyses.

**Fig. 7:**
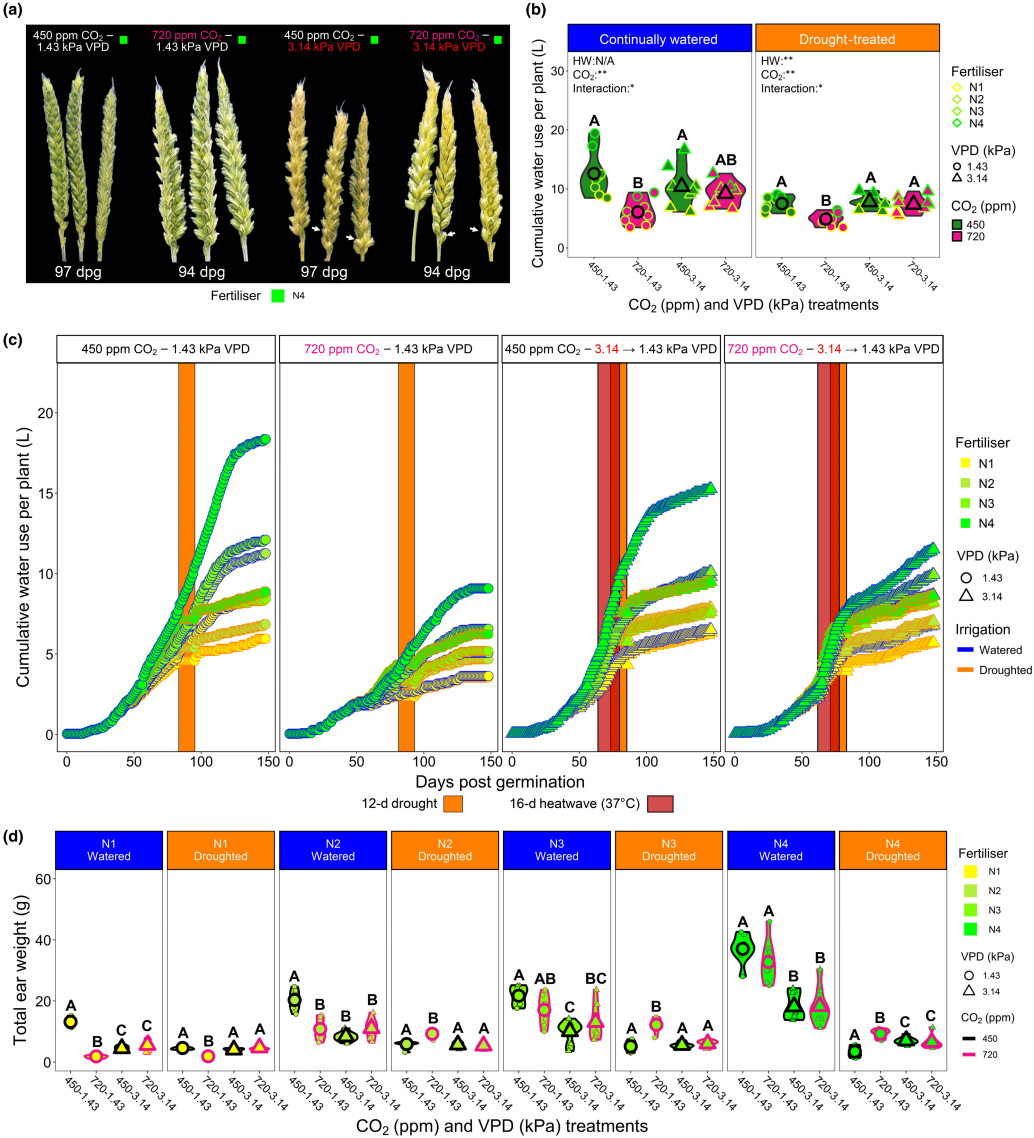
Impacts of high CO_2_, heatwaves and nitrogen (N) fertiliser application on wheat seasonal water usage and productivity. (a) Photographic images of wheat ears postheatwave and drought treatment. *Note that bulbous grains (white arrows) developed on wheat ears that experienced high vapour pressure deficit (VPD) heatwave treatments, but not on non‐heatwave ears. (b) Quantified total water usage and (c) water usage throughout a 148‐day growing season for four different CO_2_–VPD treatment combinations. (d) Total ear weight of well‐watered and droughted plants. For (b, d), *n* = 8; for c, *n* = 2. Larger symbols equal sample means. In (b), two separate two‐way ANOVAs were conducted followed by Tukey *post hoc* tests. Two separate three‐way generalised linear models (GLMs) were employed in (d), one for continually watered plants, and one for droughted plants, with estimated marginal means computed to probe for differences within N‐fertiliser groupings, with the cld function and Sidak adjustment applied to detect significance between growth treatments. Within individual graphs, different letters indicate significant differences of *P <* 0.05. *, *P* < 0.05; **, *P <* 0.01; ***, *P* < 0.001. See Supporting Information Tables S25 and S26, for statistical information relating to the significance of treatments and potential interactions for three‐way GLM analyses.

Removing irrigation as wheat ears were emerging from canopies revealed marked differences in drought stress resilience, dependent on CO_2_ and VPD treatment (Figs 6, 7, S12, S13; Tables S21–S24). Nonheatwave, high‐CO_2_ plants often performed best under drought conditions, with two out of four N‐fertiliser treatments (N2 and N3) maintaining significantly higher *E* (three‐way GLM, EMM with cld function and Sidak adjustment, *P* ≤ 0.01; Fig. 6a–c) and three out of four N‐fertiliser treatments (N2–N4) having significantly higher MTCI (three‐way GLM, EMM with cld function and Sidak adjustment, *P* < 0.0001; Fig. S12). By drought day 12, water was mainly absent from the apical portions of most wheat canopies. For N4 plants across different CO_2_ and VPD treatments, this led to whitening of wheat ears, except for nonheatwave, high‐CO_2_ plants, which still had green ears (Figs 6d, S13). This led to significantly warmer ear temperatures for the high‐CO_2_ nonheatwave plants, due to a greater proportion of incoming radiation being reflected from desiccated ears, which made them cooler (two‐way ANOVA, Tukey's HSD, *P* ≤ 0.01; Fig. 6d,e).

Heatwaves increased the speed of wheat development, which led to faster declines in leaf *E* and MTCI values (Figs 6, S12). This quicker maturation was also seen during ear and grain development, with ears that developed under heatwaves quicker to develop bulbous grains (Fig. 7a). Assessment of total water application over the 148‐d duration of experiments confirmed nonheatwave, high‐CO_2_ plants used *c*. 52% less water per plant (6.09 l) than ambient CO_2_ plants (12.6 l) when continually watered (two‐way ANOVA, Tukey's HSD, *P* < 0.001; Fig. 7b,c). There were no significant differences in total water application between CO_2_ treatments for continually watered or droughted plant comparisons for plants exposed to high‐VPD heatwaves; although under well‐watered conditions, N4 ambient CO_2_ heatwave plants did exhibit a trend towards requiring more water than N4 high‐CO_2_ heatwave equivalent plants (two‐way ANOVA, Tukey HSD, *P* = 0.19; Fig. 7b,c).

N‐fertiliser was a crucial determinant of ear weight and total aboveground biomass (a proxy for yield; Figs 7c,d, S14a; Tables S25–S28). Nonheatwave, high‐CO_2_ plants almost always produced the highest total ear weight during drought (N2–N4 N‐fertiliser treatments), but this was not the case for total aboveground biomass accumulation (Figs 7c, S14a). High‐VPD heatwaves impacted ear weight and total biomass the most in highly N‐fertilised plants, and this typically occurred irrespective of CO_2_ growth conditions. Assessment of WUE (total ear weight/ total water applied) over the whole lifecycle revealed highly fertilised N4 wheat grown under nonheatwave, high‐CO_2_ conditions had the highest WUE, requiring 286 ml of water for every gram of ear, compared with 501 ml for ambient CO_2_ plants (three‐way GLM, EMM with cld function and Sidak adjustment, *P* < 0.001; Fig. S14b; Tables S29, S30). High‐VPD heatwaves reduced overall WUE, and although the mean values of high‐CO_2_‐grown plants (676 ml) were lower than the ambient CO_2_ equivalent plants (867 ml), this was not statistically significant (three‐way GLM, EMM with cld function and Sidak adjustment, *P* = 0.44; Fig. S14b).

## Discussion

### Wheat physiological responses to current and future heatwave conditions

**Fig. 8:**
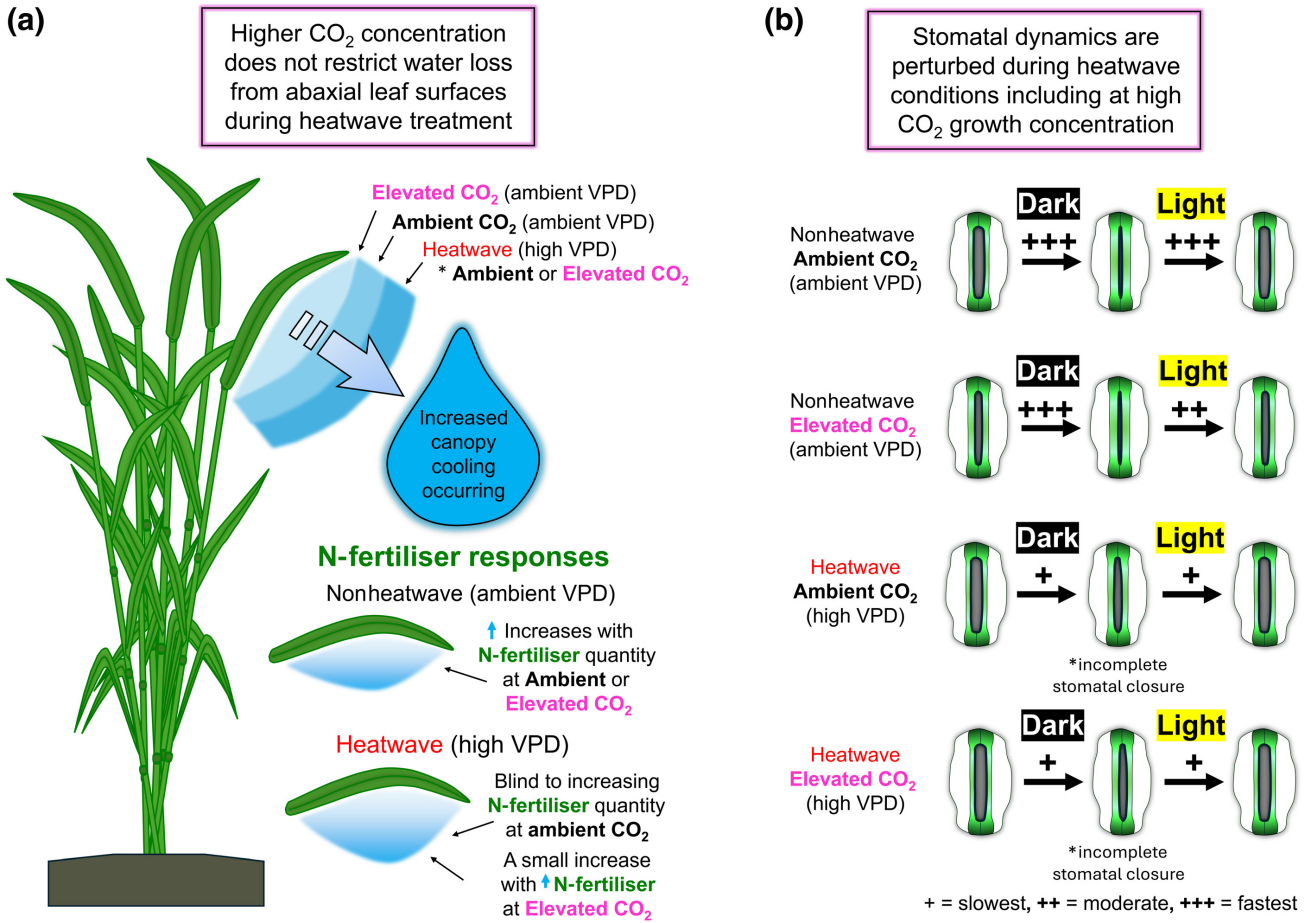
Water saving traits commonly associated with rising atmospheric CO_2_ concentration are diminished when wheat is exposed to high vapour pressure deficit (VPD) heatwaves. (a) The abaxial leaf surface contribution to plant water release and evaporative cooling is dependent on atmospheric CO_2_ concentration, heatwave application (increased VPD) and nitrogen (N) fertiliser application. (b) Wheat stomatal dynamics in response to irradiance changes are perturbed during heatwaves irrespective of CO_2_ concentration.

A robust understanding of how cultivated crops respond to environmental extremes will be crucial for developing future climate resilience. Wall *et al*. (2022) previously highlighted the dominant effect of the wheat adaxial leaf surface on gaseous exchanges, showing adaxial leaf *A* and *g*_sw_ contributions to be approximately twice that of abaxial leaf surfaces. Here, by assaying both wheat leaf surfaces within actively growing canopies, we highlight how abaxial and adaxial leaf surface contributions to *g*_sw_ and *E* depend on multiple factors including: the concentration of atmospheric CO_2_, N‐fertiliser quantity and the prevailing VPD growth conditions (Figs 3, 4, S7–S8). Higher CO_2_ concentrations typically restrict *E*/*g*_sw_ under ambient VPD, whereas higher N‐fertiliser application leads to the opposite response. During high‐VPD heatwaves, both CO_2_ and N‐fertiliser stomatal responses become diminished or attenuated, with higher evaporative water release from abaxial leaf surfaces increasingly important for canopy cooling (Fig. 8a). Overall, heatwave‐driven ‘stomatal blindness’ to higher CO_2_ concentration diminished the positive drought responses often associated with growth at higher CO_2_ (Fig. 6). This led to droughted wheat ear weights of high CO_2_ and ambient CO_2_ heatwave plants not being significantly different (Fig. 7).

Previous research conducted on fertilised wheat grown at high‐CO_2_ concentration revealed *A*_sat_ values that were typically higher, and *g*_sat_ values that were typically lower than ambient CO_2_ grown equivalent plants, leading to an overall increase in iWUE for high‐CO_2_ plants when measured under ambient growing temperature and VPD (Abdelhakim *et al*., 2022; Chavan *et al*., 2022). During heatwave conditions (higher temperature but same RH%), *A*_sat_ values again were often found to be higher for high‐CO_2_ grown plants than for ambient CO_2_ equivalent plants, but *g*_sat_ values were not significantly different in three out of the four wheat cultivars measured, revealing that like in our experiments, iWUE differences between ambient and high‐CO_2_‐grown plants often reduce (but are still significant), when elevated and ambient CO_2_ treatments are compared under high‐VPD heatwave conditions (Abdelhakim *et al*., 2022; Chavan *et al*., 2022). These experiments also found that high temperature and VPD nullified any yield increases that were routinely detectable for high‐CO_2_‐grown wheat that was cultivated under ambient growth temperatures.

There is limited research covering the combined effects of elevated CO_2_ concentration, high VPD, temperature and N deposition on crop gaseous exchanges and productivity, and so climate models are currently limited with regard to parametrisations that combine these four key growth factors. High CO_2_ or high VPD often reduce *g*_sw_ (Chater *et al*., 2015; Grossiord *et al*., 2020; Koolmeister *et al*., 2024; Slot *et al*., 2024), whereas the interactive effects of both factors have sometimes been shown to limit such decreases (Jiao *et al*., 2019; Vanaja *et al*., 2024) and a recent global meta‐analysis suggests that the combined effects of high N deposition and rising temperature oppose *g*_sw_ reductions (Liang *et al*., 2023). Here, we show during high‐VPD heatwave treatments (with moderately high temperature), stomatal light (Figs 5, 8b) and CO_2_ responses (Fig. S11) of *g*_sw_ are diminished (or absent) in high N‐fertilised plants. Such responses prompted a change in optimal stomatal behaviour (Medlyn *et al*., 2011) to favour increased water release (Fig. 1), and for high‐CO_2_ plants, this may have also been linked with greater nutrient requirements to support higher *A*, with Chl biosynthesis clearly upregulated in response to high‐VPD heatwave treatment (Figs 1a,b, 2a,b). As *D* ^0.8^ enabled a better fit than √*D* for modelling *g*_sat_ using SO theory, the data suggest that stomata were less sensitive to VPD than might be expected by the original SO model. This adds to the existing literature highlighting that stomatal VPD responses vary between species and environments (Slot *et al*., 2024). Whilst positive for wheat cooling, such a strategy has risks, especially if irrigation is limited. Despite lower *A*_sat_ values, ambient CO_2_ heatwave plants maintained similar *g*_sat_ and *E*_sat_ to high CO_2_ heatwave plants, which implies decoupling between *A*_sat_ and *g*_sat_ was occurring as higher water fluxes were still essential for apical leaves. Given the substantial reductions in ear weight of all high‐VPD heatwave plants, the maintained high water release may have been an attempt to mitigate against the impacts of temperature‐induced infertility during flowering, which is known to reduce grain yields by *c*. 3–5% for every 1 degree above optimum temperatures (Prasad & Djanaguiraman, 2014).

Irrespective of CO_2_ concentration, N1 plants greatly increased leaf *g*_sw_ and *E* to maintain evaporative cooling during heatwaves (Figs 3, 4, S6, S7). This highlights a concerning scenario for geographical areas where N‐fertiliser and irrigation are limited and/or will be limited in future (this includes many countries of the global south). To build resilience in such regions, enhanced irrigation strategies will be required so that when heatwaves occur, the rapid onset of drought can be prevented.

Greater N stimulates Chl production (Fig. S4a), promotes tillering and increases higher leaf area index (Bauer & von Wirén, 2020; Caine *et al*., 2024), which in our experiments translated into higher biomass and yields (Figs 7, S14a). We observed that N also impacts the thermal properties within wheat canopies, with highly fertilised N plants often having more leaves and visibly cooler canopies (compare different N canopies in Figs 4a, S6k). As higher N promotes more humid canopies (Azam *et al*., 2024), this would have likely led to considerably different microclimates for gaseous exchanges between our N treatments, which for high‐N plants would have likely lowered VPD conditions, especially in highly shaded areas of denser canopies. Whilst dense crop growth typically provides cooling benefits, it can also reduce CO_2_ concentration within canopy spaces (Ney & Graf, 2018; Hidaka *et al*., 2022). In our experiments, this could have led to high‐N plants having microclimate pockets with lower concentrations of CO_2_ within the canopy, especially if mixing of air was insufficient amongst dense vegetation. Such trade‐offs between enhanced cooling potential and reductions in CO_2_ availability need to be studied across different N treatments, and under field conditions, if we are to truly understand how our future crops will perform in response to the combination of higher CO_2_ concentration and increased VPD.

### Cellular and molecular considerations for optimising stomatal gas exchange during future climates

High‐VPD‐driven differences in stomatal responsiveness in our experiments occurred alongside differences in SS and SD, with heatwave imposition and higher CO_2_ both contributing towards larger SS and smaller SD, but only on abaxial leaf surfaces (Fig. 5). Large SS and small SD responses to elevated CO_2_ are also detectable in a range of different plant species, including fossilised leaf samples, but in some wheat varieties and in other examples similar SS and SD responses do not occur (Franks & Beerling, 2009; Franks *et al*., 2012; Haworth *et al*., 2023; Wall *et al*., 2023). When SS increases and SD decreases, a reduction in calculated maximum stomatal conductance (*g*_smax_) occurs, and because *g*_smax_ can be positively correlated with *g*_sw_ (Franks & Beerling, 2009; Bertolino *et al*., 2019), lower *g*_smax_ may be beneficial for saving water under steady‐state conditions due to lower *g*_sw_. However, in natural rice varieties with lower *g*_smax_ (where plants had larger SS and smaller SD), higher *E* was detected during increasing VPD treatment (without reducing *g*_sw_), whereas for small SS, high SD rice plants (with higher *g*_smax_), *g*_sw_ reductions restricted *E*, with *A* markedly reduced as a consequence (Caine *et al*., 2023). Smaller stomata are proposed to enable quicker responsiveness to environmental stimuli because of greater surface area to volume ratios (Hetherington & Woodward, 2003; Drake *et al*., 2013; Raven, 2014) and coupled with higher SD, this has been suggested to also boost carbon uptake via higher *g*_smax_ when atmospheric CO_2_ concentration is low (Franks & Beerling, 2009). If the need to release water at moderately high VPD in future climates overrides selective pressures to close stomata in the presence of lower light (or higher CO_2_), then having overly responsive small SS might limit plant performance. If this is the case, then optimising stomatal *g*_sw_, via smaller SS and larger SD (for enhanced iWUE under dynamic light situations), might also require stomata to remain open during incidences of higher VPD. To produce this additional responsivity, alterations to both stomatal development and function may well be required.

Research in *Arabidopsis thaliana* (Arabidopsis) and in crops is highlighting some of the core molecular mechanisms that alter stomatal development and function in response to N availability, CO_2_ concentration, higher temperature, higher VPD and drought (Hashimoto *et al*., 2006; Engineer *et al*., 2014; Lau *et al*., 2018; Mohamed *et al*., 2023; Koolmeister *et al*., 2024; Pankasem *et al*., 2024; Wang *et al*., 2025; Xu *et al*., 2025). In wheat, it is currently unclear how stomatal development is regulated in response to all these different environmental stimuli concurrently, but genes orthologous to the *EPIDERMAL PATTERNING FACTOR* (*EPF*) and *EPF‐Like* (*EFPL*) genes are present in wheat, which encode small signalling peptides that regulate stomatal patterning and stress responsiveness (Hepworth *et al*., 2018; Dunn *et al*., 2019; Mohamed *et al*., 2023; Kim & Torii, 2024). In Arabidopsis, stomatal patterning is negatively regulated by EPF1 and EPF2, which function to repress stomatal development leading to lower SD. Opposingly, EPFL9 (otherwise known as STOMAGEN) competes with EPF1/2 to promote stomatal development and increase SD (Kim & Torii, 2024). Research in Arabidopsis shows that SS and SD display an inverse relationship when *EPF/L* genes are knocked out and/or overexpressed (Doheny‐Adams *et al*., 2012), suggesting that EPF/L gene targets could be good options for altering the SS‐SD relationship in crops. EPF/L‐driven SS and SD alterations have now been partially explored in rice, but it seems so far that the inverse relationship may be cultivar‐specific when *EPF/L* gene expression is manipulated (Caine *et al*., 2019; Mohammed *et al*., 2019). It is unclear whether overexpression of *EPFL9* (or the grass‐specific *EPFL10*) will reduce SS and increase SD in wheat, but if this does occur, this could provide a valuable tool for enhancing WUE under dynamic light situations by generating faster stomatal responsivity. Such alterations could provide a building block for generating the multiresponsive stomata required for growth under future climates, but maintained opening during high VPD will also be required for maintaining photosynthetic productivity.

Regulating stomatal pore aperture in response to rising CO_2_ concentration, temperature and VPD is complex, and research in Arabidopsis and wheat shows that the receptor kinase OPEN STOMATA 1 (OST‐1) and the S‐type anion channel SLOW ANION CHANNEL‐ASSOCIATED 1 (SLAC1) are particularly important for integrating the aforementioned environmental stimuli (Tian *et al*., 2015; Tulva *et al*., 2023; Takahashi *et al*., 2025; Xu *et al*., 2025). As stomatal N responses were reduced or lost during our heatwave experiments (Figs 3, 4), we will focus here mainly on high CO_2_, temperature and VPD stomatal aperture regulation and will also consider drought where appropriate. Recently, OST‐1 has been shown to phosphorylate the mitogen‐activated protein kinase kinase kinase kinase (MAP4K4) TARGET OF TEMPERATURE 3 (TOT3) in GCs, leading to its inactivation, which prevents stomatal opening during drought (Xu *et al*., 2025). On the other hand, when temperature (and VPD) is higher, TOT3 is triggered, activating the GC plasma membrane‐bound H^+^‐ATPase 1 (AHA1), which facilitates stomatal opening, but this heat activation of TOT3 is tempered when drought is also present (Xu *et al*., 2025). Assessment of Arabidopsis *ost1‐*3 mutants grown under well‐watered conditions has revealed that *g*_sw_ remained higher than in col‐0 wild‐type plants during high temperature/VPD (Tulva *et al*., 2023), implying that OST‐1 also contributes to stomatal aperture responsiveness to high heat in addition to its regulation of TOT3 during drought.

In Arabidopsis, the CO_2_‐responsive Raf‐like kinase HIGH TEMPERATURE 1 (HT1) enables stomatal opening at lower CO_2_ concentrations by phosphorylating the Raf‐like kinase CONVERGENCE OF BLUE LIGHT AND CO_2_ 1 (CBC1), which prevents an unknown downstream intermediate protein from phosphorylating SLAC1 (Takahashi *et al*., 2022, 2025). This prevents the efflux of anions by SLAC1 and so GC turgor does not reduce, and stomata are prevented from narrowing. When CO_2_ concentration is elevated, increases in bicarbonate within GCs lead to HT1 being sequestered by MITOGEN‐ACTIVATED KINASES (MPK) 4 and MPK12, which prevents CBC1 from inhibiting the unknown downstream intermediate protein, and so SLAC1 does become phosphorylated and stomata begin to close as GC turgor decreases (Takahashi *et al*., 2022, 2025). It has been suggested that when ABA is lower, OST‐1 displays some basal kinase activity, which could also phosphorylate the same unknown intermediate protein that acts downstream of CBC1, which aids stomatal closure at high CO_2_ (Takahashi *et al*., 2025). Given the key role of HT1 in regulating stomatal pore responses at high‐CO_2_ concentration, it will be interesting to next explore whether HT1 is still sequestered by MPK4/12 during high‐temperature/VPD treatment at high CO_2_, or whether HT1 remains active and can phosphorylate CBC1. If HT1 does remain active, then SLAC1 would not become phosphorylated, and this could explain why stomata remain open during high‐CO_2_ heatwaves.

### Conclusion

Taken together, our results show that wheat stomata are insensitive to CO_2_‐induced stomatal closure during high‐VPD heatwaves in the wheat variety we assessed, and this is driven by increased water fluxes from the abaxial leaf surface (Fig. 8). Such a response led to high CO_2_ grown plants using considerably more water than ambient CO_2_ plants during the initial stages of heatwaves, with this increased evaporative demand potentially linked to higher photosynthetic capacity. Stomatal adjustments in response to irradiance changes (and rising CO_2_) were also significantly impaired (Figs 8b, S11), which together suggests that future wheat could be considerably less water‐use efficient than previously envisioned. To corroborate whether the same responses occur in other wheat varieties, and to understand whether similar CO_2_–VPD responses arise in other crops, further research is urgently required so that crop resilience and global modelling efforts can be correctly optimised for future climates.

## Competing interests

None declared.

## Author contributions

RSC and HLC designed the study. RSC, MSK and YS collected the data. RSC undertook data analysis. RSC and HLC wrote the manuscript with input from MSK, YS and CPO.

## Disclaimer

The New Phytologist Foundation remains neutral with regard to jurisdictional claims in maps and in any institutional affiliations.

## Supporting information


**Fig. S1** Schematic overview of experimental design.
**Fig. S2** Stomatal optimality modelling and alterations to the *D* parameter.
**Fig. S3** Extended data for wheat saturating light gas exchange.
**Fig. S4** Extended data for wheat biochemical analyses.
**Fig. S5** Wheat CO_2_ response curve and supply function assessment.
**Fig. S6** Increased N‐fertiliser boosts wheat gaseous exchanges.
**Fig. S7** Heatwave impacts on gaseous exchanges from both leaf surfaces.
**Fig. S8** Thermal profiling and water application assessment of heatwave‐grown wheat.
**Fig. S9** Wheat gas exchange before light‐shift treatment.
**Fig. S10** Leaf temperature responses to changes in irradiance and VPD.
**Fig. S11** Wheat gas exchange responses to high CO_2_ treatment.
**Fig. S12** MERIS terrestrial Chl index (MTCI) changes during drought treatment.
**Fig. S13** Thermal profiling of high N‐fertilised wheat during drought and heatwave treatment.
**Fig. S14** Aboveground biomass and whole‐plant water‐use efficiency (WUE) over the entirety of growth experiment.
**Tables S1–S30** Statistical analysis tables highlighting wheat responses to different VPD, CO_2_ growth concentration, N‐fertiliser and drought treatments.

**Dataset S1** Data quantified within the manuscript.

**Dataset S2** Hyperspectral data of flag leaves pre‐drought.

**Dataset S3** Hyperspectral data of flag leaves of plants grown at 25°C.

**Dataset S4** Hyperspectral data of flag leaves during drought.
Please note: Wiley is not responsible for the content or functionality of any Supporting Information supplied by the authors. Any queries (other than missing material) should be directed to the *New Phytologist* Central Office.

## Data Availability

The data assessed within this manuscript are provided in Supporting Information Datasets [Link], [Link], [Link], [Link].
