# Supplementary material for: Future heatwave conditions inhibit CO2 ‐induced stomatal closure in wheat
Source: New Phytol. 2025 Nov 16;249(3):1234–52. doi: 10.1111/nph.70722 (PMC12780326; doi:10.1111/nph.70722)
Supplement: Supplementary file 1 — Fig. S1 Schematic overview of experimental design. Fig. S2 Stomatal optimality modelling and alterations to the D parameter. Fig. S3 Extended data for wheat saturating light gas exchange. Fig. S4 Extended data for wheat biochemical analyses. Fig. S5 Wheat CO2 response curve and supply function assessment. Fig. S6 Increased N‐fertiliser boosts wheat gaseous exchanges. Fig. S7 Heatwave impacts on gaseous exchanges from both leaf surfaces. Fig. S8 Thermal profiling and water application assessment of heatwave‐grown wheat. Fig. S9 Wheat gas exchange before light‐shift treatment. Fig. S10 Leaf temperature responses to changes in irradiance and VPD. Fig. S11 Wheat gas exchange responses to high CO2 treatment. Fig. S12 MERIS terrestrial Chl index (MTCI) changes during drought treatment. Fig. S13 Thermal profiling of high N‐fertilised wheat during drought and heatwave treatment. Fig. S14 Aboveground biomass and whole‐plant water‐use efficiency (WUE) over the entirety of growth experiment. Tables S1–S30 Statistical analysis tables highlighting wheat responses to different VPD, CO2 growth concentration, N‐fertiliser and drought treatments. [file NPH-249-1234-s001.pdf]

New Phytologist Supporting Information

Future heatwave conditions inhibit CO_2-induced stomatal closure in wheat

Robert S. Caine[1,2]✉, Muhammad S. Khan[1], Yixiang Shan[1], Colin P. Osborne[1,2], Holly L. Croft[1,2]✉

[1]Plants, Photosynthesis and Soil, School of Biosciences, University of Sheffield, South Yorkshire S10 2TN, UK.

[2]Institute for Sustainable Food, School of Biosciences, University of Sheffield, South Yorkshire, S10 2TN, UK.

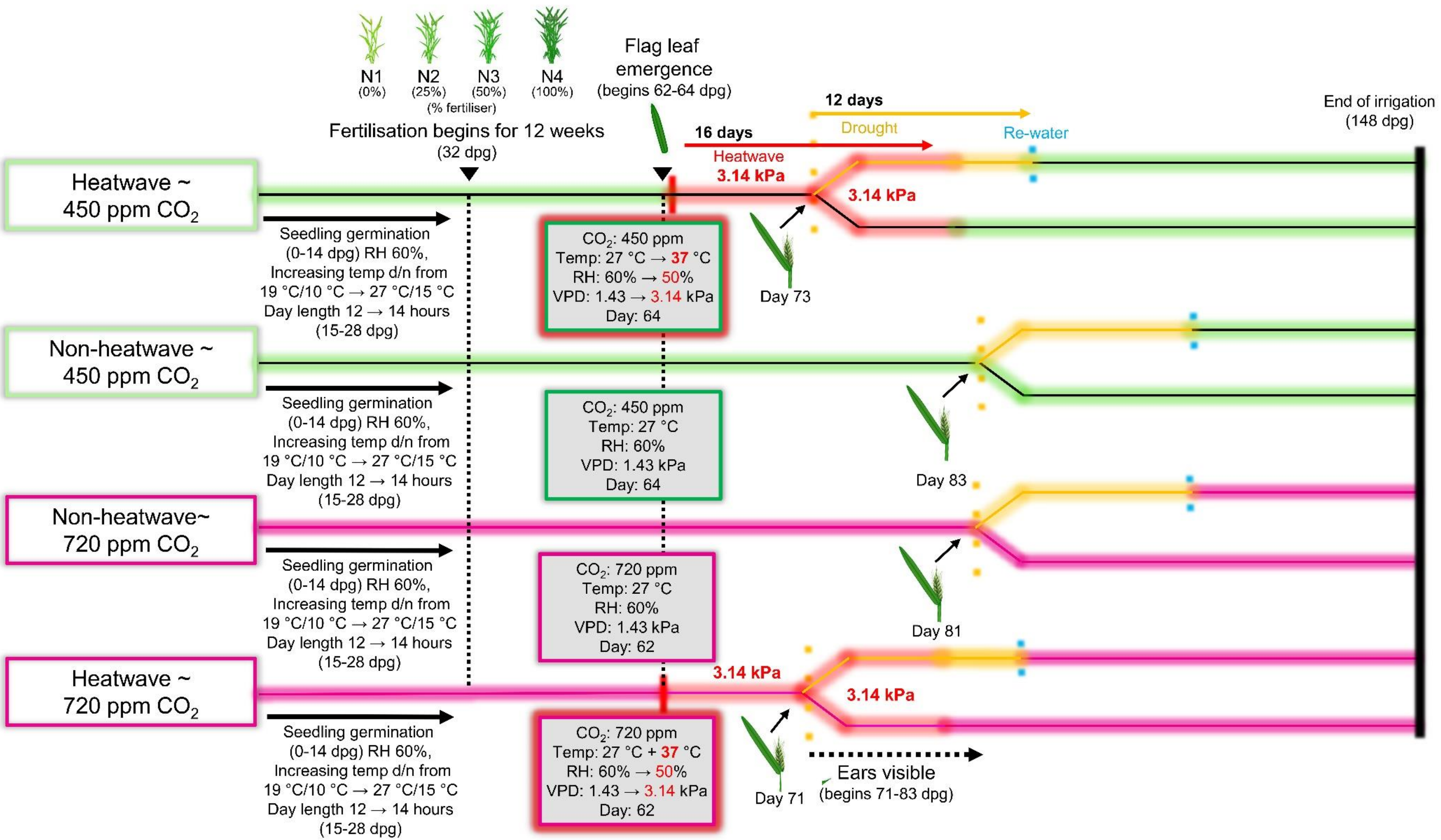

Fig. S1: Simulating future environmental extremes to study wheat responses to rising CO_2 concentration and high vapour pressure deficit (VPD) heatwaves. Wheat was cultivated at either 450 ppm or 720 ppm CO_2 concentration throughout and grown under either temperate or

heatwave conditions. From day 0 to 14 plants, all 4 CO_2~VPD treatments (heatwave/ non-heatwave) were grown at 19 °C at the canopy (including +4°C from lights) with RH set to 60%. From days 15-28, the temperature was gradually increased from 19 °C to 27 °C during the day (including +4°C from lights) and from 10 °C to 15 °C during the night. During this time the day length was also gradually increased from 12 to 14 hours. By 28 the VPD was set to 1.43 kPa (27 °C, 60% relative humidity) for all treatments. At day 32, a 12-week nitrogen (N) fertiliser treatment was started (N1-N4). The range of high N-fertiliser treatments were included within each growth scenario (N1 = 0%, N2 = 25% N3 = 50% and N4 = 100% N-fertiliser application). For further details on N application see the plant materials and growth conditions sections of the method. At day 62, one set of 720ppm CO_2 plants began the heatwave treatment with the VPD increased from 1.43 kPa to 3.14 kPa (37 °C, 50% relative humidity). This was followed with the same increase in VPD for one set of 450 ppm CO2 plants at day 64. In both cases, this was when flag leaves first began to appear (for non-heatwave plants this was around the same time). The heatwave treatments were applied for 16 days, with a 12-day drought period imposed from 9 day into the heatwave treatment, as wheat ears were emerging. For the experiments where heatwaves were not applied, wheat ears emerged later, which for 720 ppm CO_2 plants was at day 81, and for 450 ppm CO_2 plants, was at day 83. All plants that missed N-fertiliser treatment during drought received the missed allocation once watering had recommenced. $n = 32$ per each N-fertiliser treatment per CO_2~VPD scenario (128 plants per each of the 4 experiments), reducing to $n = 16$ during drought.

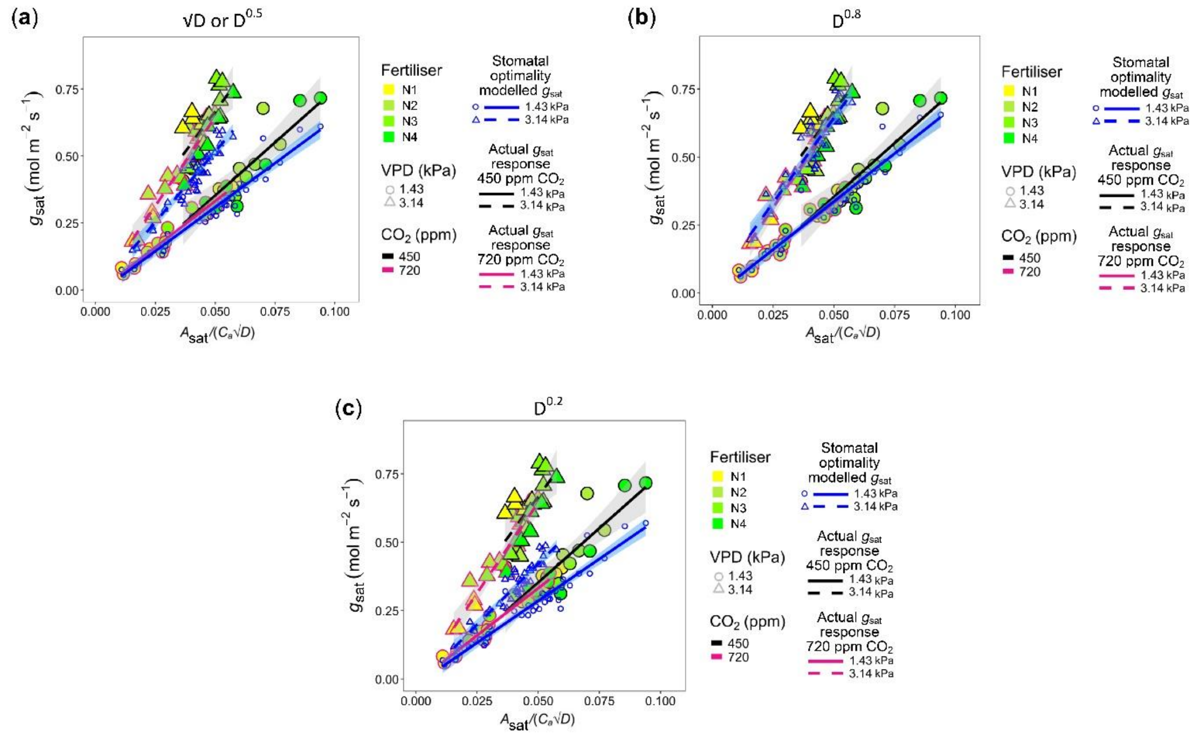

Fig. S2. Stomatal Optimality (SO) model variation based on alterations to the leaf to air vapour pressured deficit (*D*) parameter. (**a-c**) SO modelled and actual light saturated gas exchange data under differing nitrogen (N) fertiliser, CO_2 concentration and vapour pressure deficit (VPD) conditions where the parameterisation of D within g_1 of the SO model (blue data) has been adjusted in each sequential graph. In **a**, SO was modelled using a g_1 D value of $\sqrt{D}$, which also equates to $D^{0.5}$. In **b**, the D of g_1 is set to $D^{0.8}$ (as in **Fig. 1a**) and in **c**, D is set to $D^{0.2}$. The y-axis represents stomatal conductance (g_{sat}) in each graph. The x-axis, in each graph represents a measure of photosynthetic output based on the saturating light photosynthesis (A_{sat}), divided by the ambient CO_2 concentration of the air (C_a) multiplied by √D. Solid blue lines represent SO modelled g_{sat} data at 1.43 kPa VPD (non-heatwave) and dashed blue lines represents SO modelled data g_{sat} at 3.14 kPa (heatwave). The small blue symbols represent individual SO modelled values. Black (450 ppm) and pink (720 ppm) solid and dashed lines and symbols represent regression analyses alongside measured values. For all panels n = 4-5.

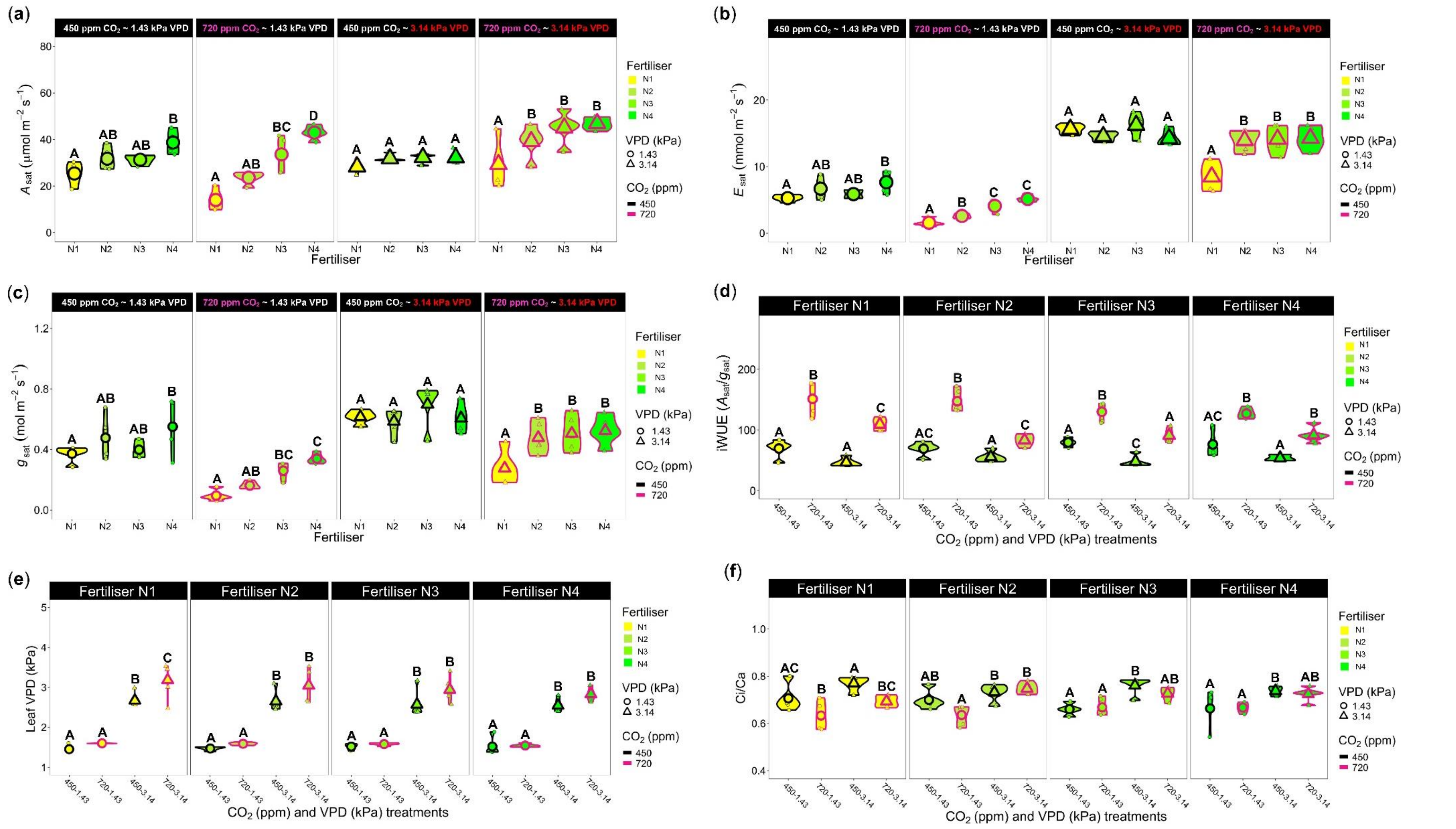

Fig. S3. Further Saturating light gas exchange analysis of wheat grown under different CO_2 concentrations and heatwave scenarios

(**a-c**) Saturating light (**a**) photosynthesis (A_{sat}) and (**b**) transpiration (E_{sat}) and (**c**) stomatal conductance (g_{sat}) grouped by CO_2~vapour pressure deficit (VPD) treatment. (**d-f**) Intrinsic water-use efficiency (iWUE; A_{sat}/g_{sat}), (**e**) Leaf VPD and (**f**) equivalent intercellular CO_2 concentration grouped by nitrogen (N) fertiliser treatment. In **a-d** and **f**, three-way ANOVAs were undertaken. For **e,** a generalised linear model (GLM) was employed. Estimated marginal means were computed to probe for differences within each graph, with the cld function and Sidak adjustment applied to detect significance between the different treatments. $n = 4\text{-}5$. Large symbols equal sample means. Within individual graphs, different letters indicate significant differences of $p \leq 0.05$. See Tables S1-S7 for statistical information relating to the significance of treatments and potential interactions for three-way ANOVA and GLM analyses.

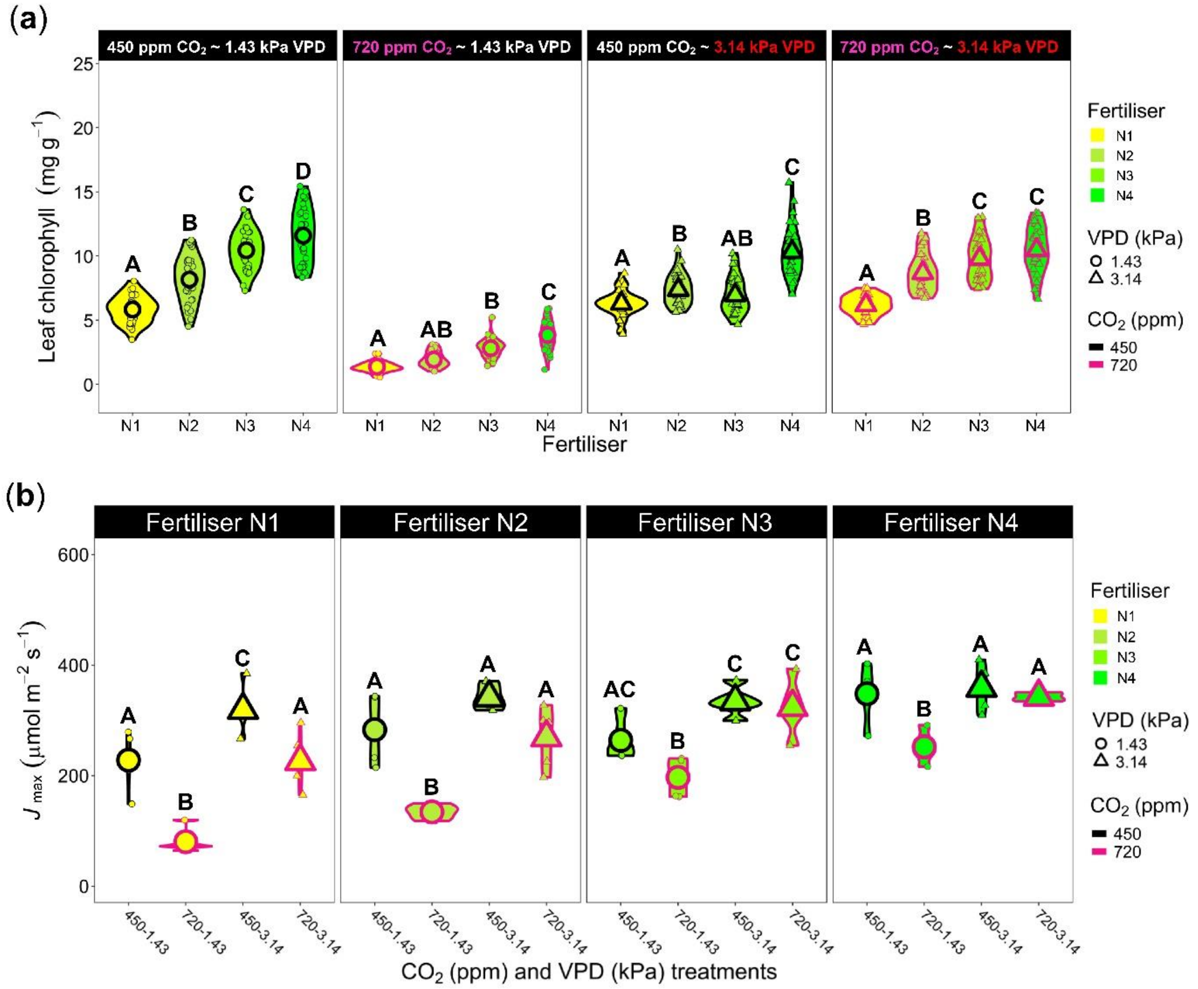

Fig. S4. Biochemical analyses of wheat assessed under different CO_2 and vapour pressure deficit (VPD) growth conditions. (**a**) Leaf chlorophyll content normalised by mass of plants growing under different CO_2 concentration, VPD and nitrogen (N) fertiliser treatments grouped via growth scenario. (**b**) Maximum rate of photosynthetic electron transport (J_{max}) of plants grown and assessed at different CO_2 concentrations and VPD scenarios, grouped by fertiliser treatment. For **a,** a generalised linear model (GLM) was employed and in **b** a three-way ANOVA was undertaken. Estimated marginal means were computed to probe for differences within CO~VPD treatments **a** or N-fertiliser groupings in **b**, with the cld function and Sidak adjustment applied to detect significance between the different treatments. For a and b: n = 31-32, for c-h n = 4-5. Except in **b**, large symbols equal sample means. Within individual graphs, different letters indicate significant differences of $p \leq 0.05$. See Tables S8 and S10 for statistical information relating to the significance of treatments and potential interactions for three-way GLM and ANOVA analysis.

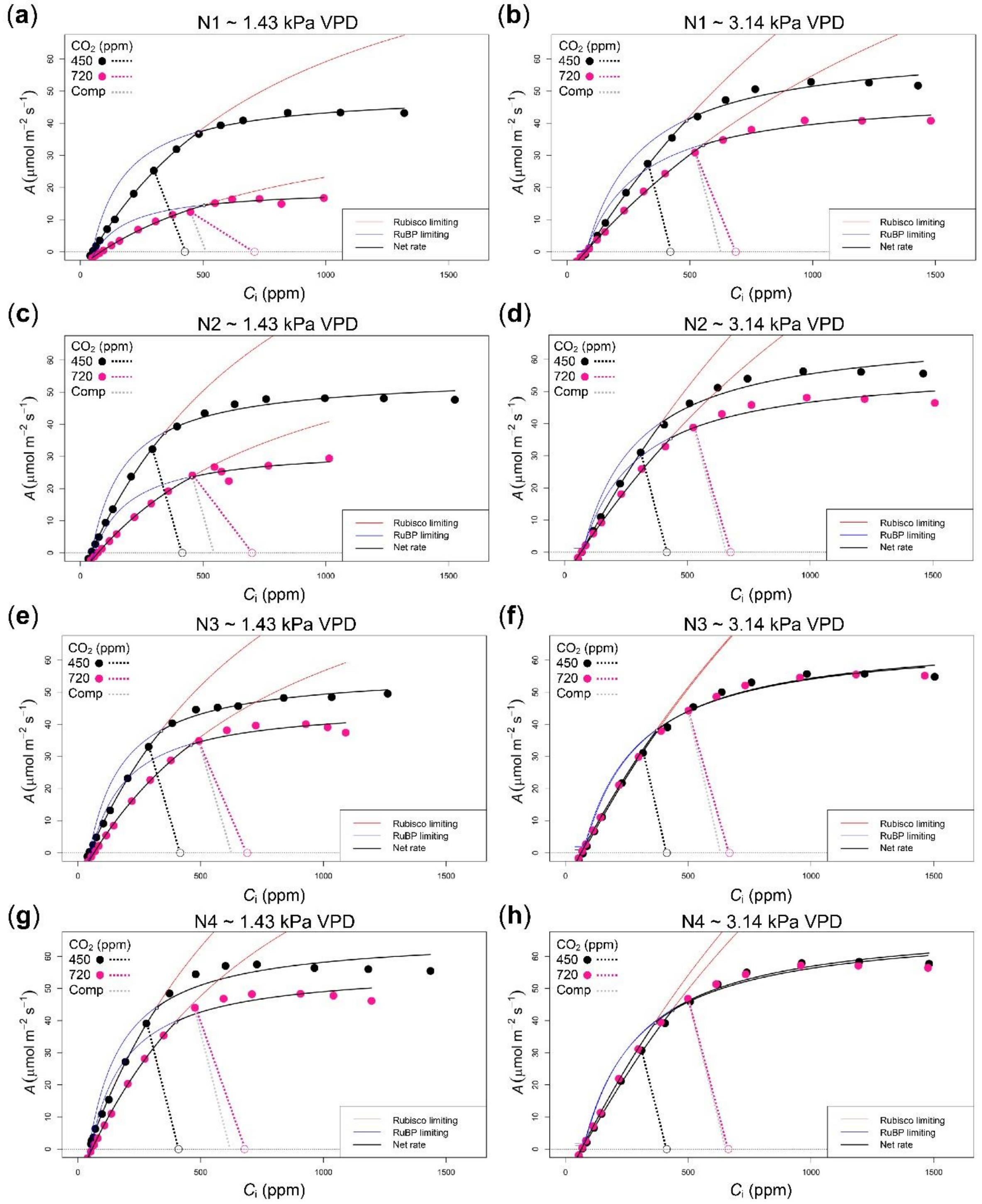

Fig. S5. Wheat CO_2 response curve and supply function assessment. (**a-h**) photosynthesis (*A*) by intercellular CO_2 concentration (C_i) curves from 4x nitrogen (N) fertiliser treatments (N1 to N4) conducted under ambient (1.34 kPa) or high vapour pressure deficit (VPD) heatwave (3.14 kPa) akin to the corresponding growth conditions of plants measured. Within each graph, supply functions (dotted lines) and data points are black for ambient CO_2 (450 ppm) treatment and pink for high CO_2 (720 ppm) treatment. Gray lines represent 450 ppm

sample slopes marked immediately adjacent to high CO_2 samples as comparisons. The hollow symbols represent C_a at growth room CO_2 concentration. n = 4-5

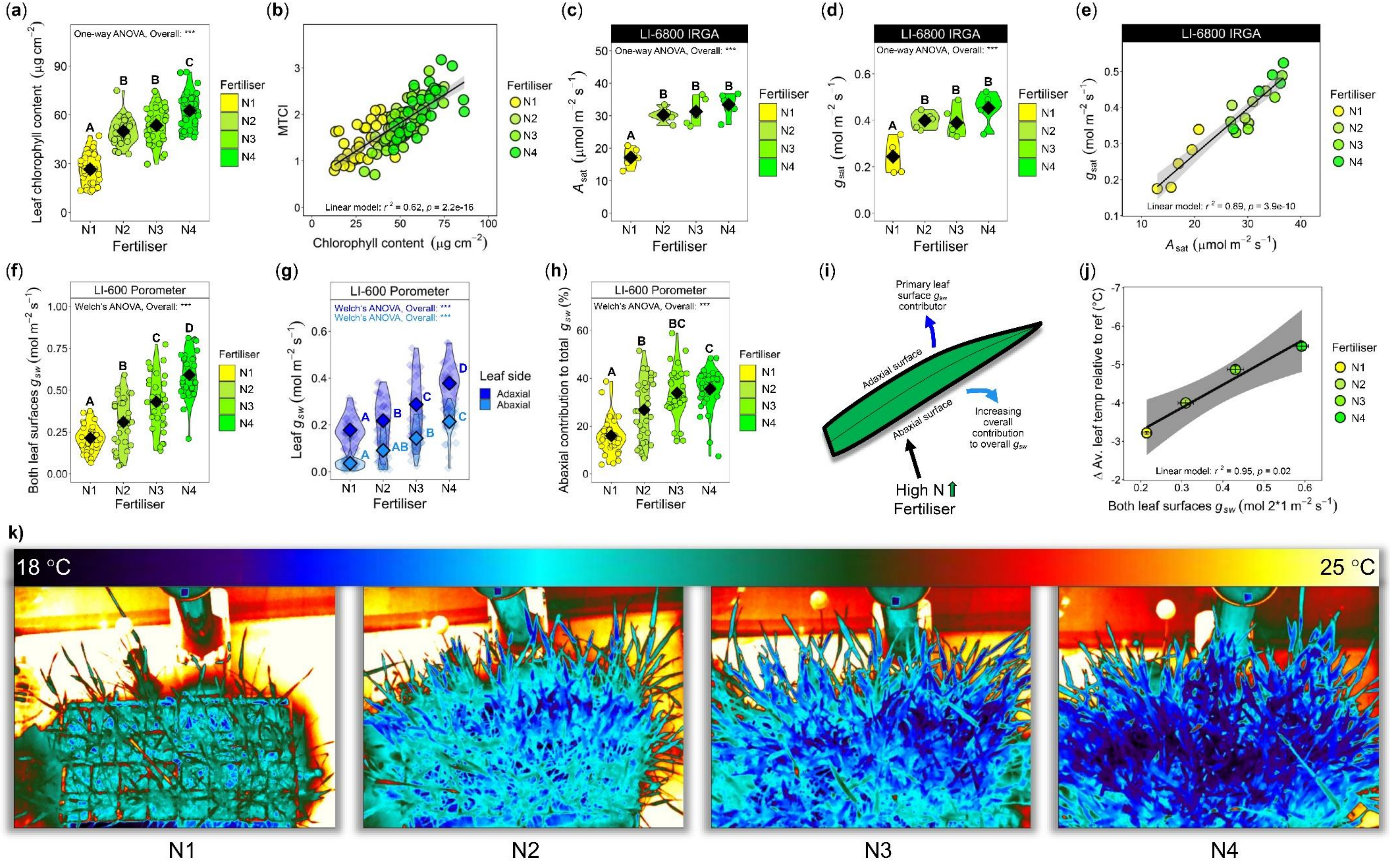

(a)
One-way ANOVA, Overall: ***
Leaf chlorophyll content (µg cm−2)
Fertiliser
N1 N2 N3 N4
(b)
MTCI
Linear model: r2 = 0.62, p = 2.2e-16
Chlorophyll content (µg cm−2)
(c)
LI-6800 IRGA
One-way ANOVA, Overall: ***
Asat (µmol m−2 s−1)
(d)
LI-6800 IRGA
One-way ANOVA, Overall: ***
gsat (mol m−2 s−1)
(e)
LI-6800 IRGA
gsat (mol m−2 s−1)
Linear model: r2 = 0.89, p = 3.9e-10
Asat (µmol m−2 s−1)
(f)
LI-600 Porometer
Welch's ANOVA, Overall: ***
Both leaf surfaces gsw (mol m−2 s−1)
(g)
LI-600 Porometer
Welch's ANOVA, Overall: ***
Welch's ANOVA, Overall: ***
Leaf gsw (mol m−2 s−1)
Leaf side
Adaxial
Abaxial
(h)
LI-600 Porometer
Welch's ANOVA, Overall: ***
Abaxial contribution to total gsw (%)
(i)
Primary leaf surface gsw contributor
Adaxial surface
Abaxial surface
Increasing overall contribution to overall gsw
High N Fertiliser
(j)
Δ Av. leaf temp relative to ref (°C)
Linear model: r2 = 0.95, p = 0.02
Both leaf surfaces gsw (mol 2*1 m−2 s−1)
k)
18 °C
25 °C
N1
N2
N3
N4

Fig. S6. Increased application of nitrogen (N) fertiliser enhances gaseous exchanges on both flag leaf surfaces of cultivated wheat. (**a**) Leaf chlorophyll content of wheat supplied with increasing concentrations of high-N fertiliser (N1 least N4 most). (**b**) MERIS terrestrial chlorophyll index (MTCI) vegetation index regressed again leaf chlorophyll content. LI-COR LI-6800 Infra-red gas analyser measurements of (**c**) saturating light photosynthesis (A_{sat}) and (**d**) saturating light stomatal conductance (g_{sat}). (**e**) Regression of g_{sat} and A_{sat}. LICOR LI-600 porometer measurements of (**f**) total g_{sw} from both leaf sides, (**g**) individual adaxial and abaxial contributions, and (**h**) abaxial % contribution to total g_{sw}. (**i**) Schematic representing N-fertiliser impacts on gaseous exchange. (**j**) Combined averaged canopy-level g_{sw} measurements of both leaf surfaces regressed against averaged Δ leaf temperature of leaves relative to an in-chamber reference surface. (**k**) Thermal images of N1-N4 wheat canopies, with thermal scale. Plants in this figure were grown as in Fig. S1 except instead of ramping to 27 °C, ramping was to 25 °C (includes +4 °C from light). All measurements were collected after flag leaves had emerged but prior to ear emergence. For all gas exchange measurements, Tair was set to 25 °C. For **a**, **b**, **f**, **g**, **h** and **j**, n = 32, for **c-e**, n = 5. Large symbols equal sample means. For one-way and two-way ANOVAs, Tukey post-hoc tests were performed to determine significance. For Welch's ANOVAs, Games-Howell post-hoc tests were undertaken. Different letters within graphs indicate significant differences of $p \leq 0.05$. Asterisks equal, * = $p < 0.05$, ** = $p < 0.01$ and *** = $p < 0.001$.

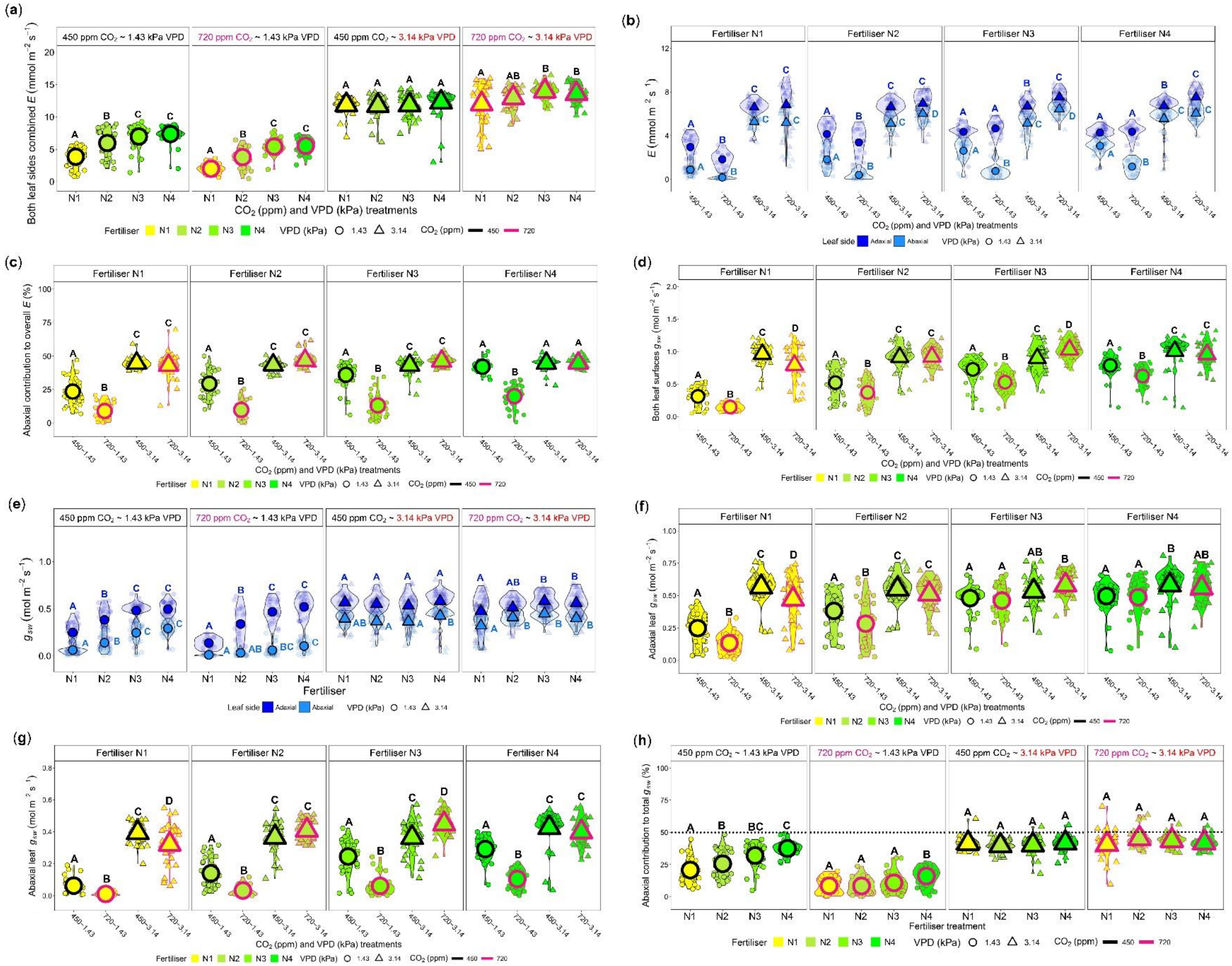
(a)
450 ppm CO2 ~ 1.43 kPa VPD
720 ppm CO2 ~ 1.43 kPa VPD
450 ppm CO2 ~ 3.14 kPa VPD
720 ppm CO2 ~ 3.14 kPa VPD
Both leaf sides combined E (mmol m−2 s−1)
CO2 (ppm) and VPD (kPa) treatments
Fertiliser N1 N2 N3 N4 VPD (kPa) 1.43 3.14 CO2 (ppm) 450 720
(b)
Fertiliser N1
Fertiliser N2
Fertiliser N3
Fertiliser N4
E (mmol m−2 s−1)
CO2 (ppm) and VPD (kPa) treatments
Leaf side Adaxial Abaxial VPD (kPa) 1.43 3.14
(c)
Abaxial contribution to overall E (%)
CO2 (ppm) and VPD (kPa) treatments
(d)
Both leaf surfaces gsw (mol m−2 s−1)
CO2 (ppm) and VPD (kPa) treatments
(e)
gsw (mol m−2 s−1)
Fertiliser
Leaf side Adaxial Abaxial VPD (kPa) 1.43 3.14
(f)
Adaxial leaf gsw (mol m−2 s−1)
CO2 (ppm) and VPD (kPa) treatments
(g)
Abaxial leaf gsw (mol m−2 s−1)
CO2 (ppm) and VPD (kPa) treatments
(h)
Abaxial contribution to total gsw (%)
Fertiliser treatment
Fertiliser N1 N2 N3 N4 VPD (kPa) 1.43 3.14 CO2 (ppm) 450 720

Fig. S7. The impacts of CO_2, vapour pressure deficit (VPD) and nitrogen (N) fertiliser treatment on wheat leaf gaseous exchanges. (**a**) Porometer measurements of transpiration (*E*) of both leaf surfaces across a gradient of nitrogen fertiliser treatments, grouped via ambient (450 ppm) or high CO_2 (720 ppm), under non-heatwave (VPD = 1.43 kPa) or heatwave (VPD = 3.14 kPa) conditions (**b**) Abaxial and adaxial *E* leaf measurements grouped by N-fertiliser treatment across growth treatment scenarios. (**c**) Abaxial leaf surface contribution to *E* grouped by N-fertiliser treatment. (**d**) Both leaf surfaces combined stomatal conductance to water vapour (g_{sw}) grouped by N-fertiliser treatment. (**e**) Abaxial and adaxial g_{sw} measurements across N-fertiliser treatment grouped by growth treatment scenarios. (**f**) Adaxial and (**g**) abaxial g_{sw} grouped via grouped by N-fertiliser treatment. (**h**) Abaxial g_{sw} contribution to total g_{sw} across N-fertiliser gradient grouped by growth scenario. For **a-g**, $n = 32$, for **h**, n = 20-32. For all data analyses, three-way generalised linear models (GLMs) were employed, with estimated marginal means computed to probe for differences within N-fertiliser groupings, or CO_2~VPD treatment combinations, with the cld function and Sidak adjustment applied to detect significance between treatments. Within individual graphs, different letters indicate significant differences of $p \leq 0.05$. See Tables S11-S18 for statistical information relating to the significance of treatments and potential interactions for three-way GLM analyses.

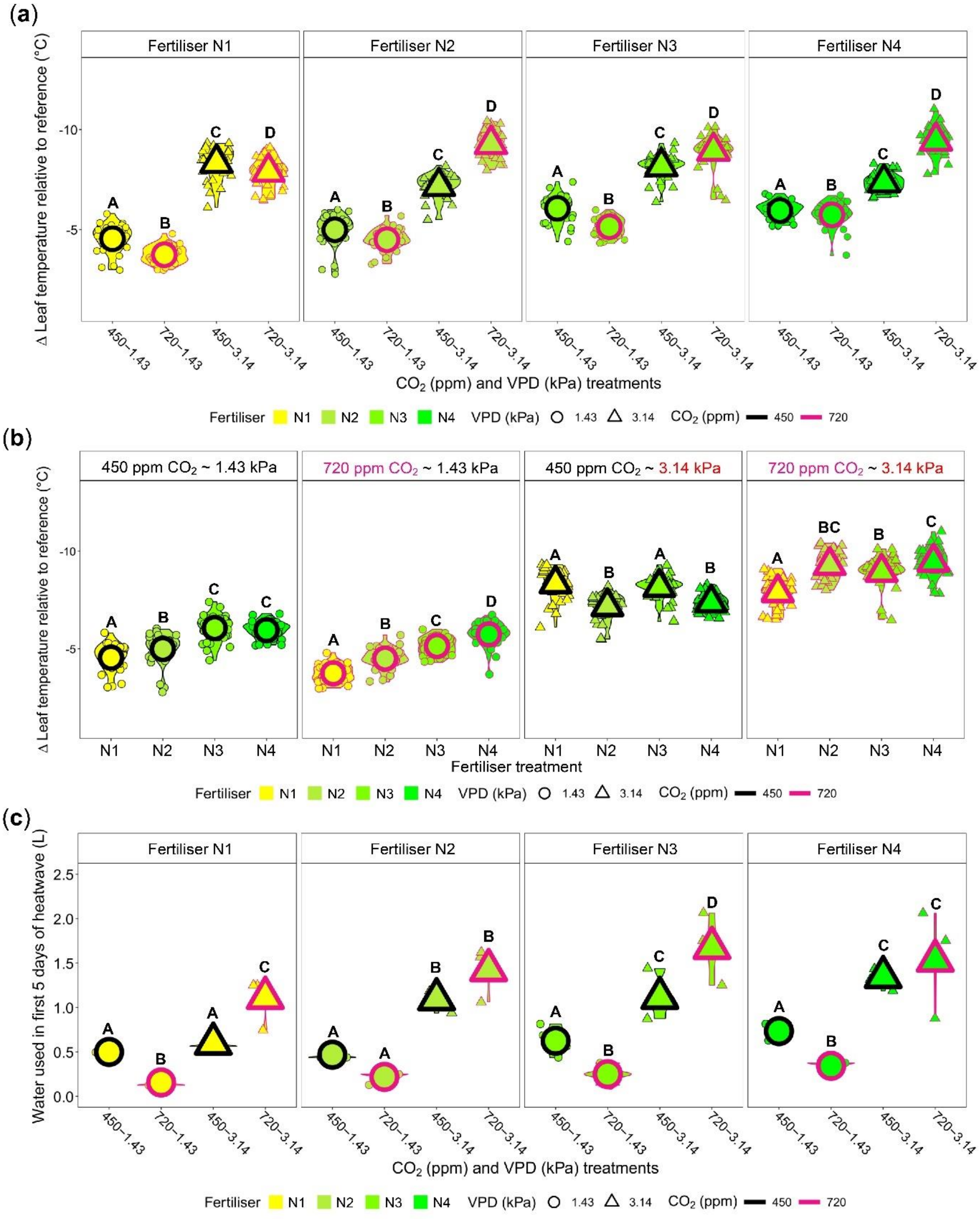

Fig. S8. Infrared thermography and whole-plant water application of wheat grown under different CO_2 and heatwave treatments. (**a**) The Δ leaf temperature differences of different CO_2 and vapour pressure deficit (VPD) treated plants, grouped by nitrogen (N) fertiliser treatment. (**b**) The Δ leaf temperature differences of different CO_2 and vapour pressure deficit treated plants, grouped by growth scenario treatment. (**c**) Water applied over the first 5 days of

heatwave. For **a** and **b**: $n = 32$, for **c**: $n = 4$. Large symbols equal sample means. For **a** and **b**, three-way generalised linear models (GLMs) were employed. For **c**, a three-way ANOVA was performed. Estimated marginal means were computed to probe for differences within N-fertiliser groupings, or CO_2~VPD treatment combinations, with the cld function and Sidak adjustment applied to detect significance between treatments. Within individual graphs, different letters indicate significant differences of $p \leq 0.05$. See Tables S19-S20 for statistical information relating to the significance of treatments and potential interactions for three-way GLM analyses.

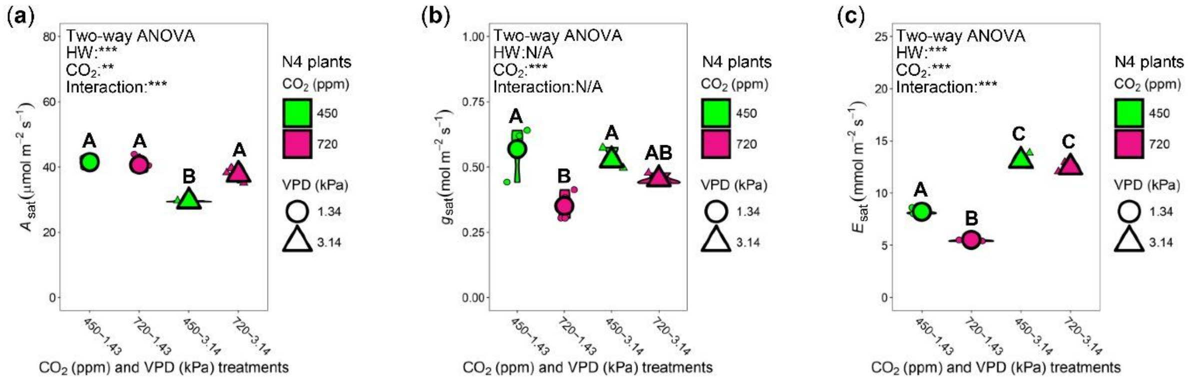

Fig. S9. Wheat gaseous exchanges under light-saturating conditions immediately prior to dark treatment. Measurements conducted under (**a-c**) Saturating light (**a**) photosynthesis (A_{sat}), (**b**) stomatal conductance to water vapour (g_{sat}) and (**c**) transpiration (E_{sat}) using corresponding growth chambers conditions to growth chambers. n = 3-4. Large symbols equal means. For Two-way ANOVAs, Tukey post-hoc tests were performed. Different letters within graphs indicate significant differences of $p \leq 0.05$. Asterisks equal, * = $p < 0.05$, ** = $p < 0.01$ and *** = $p < 0.001$.

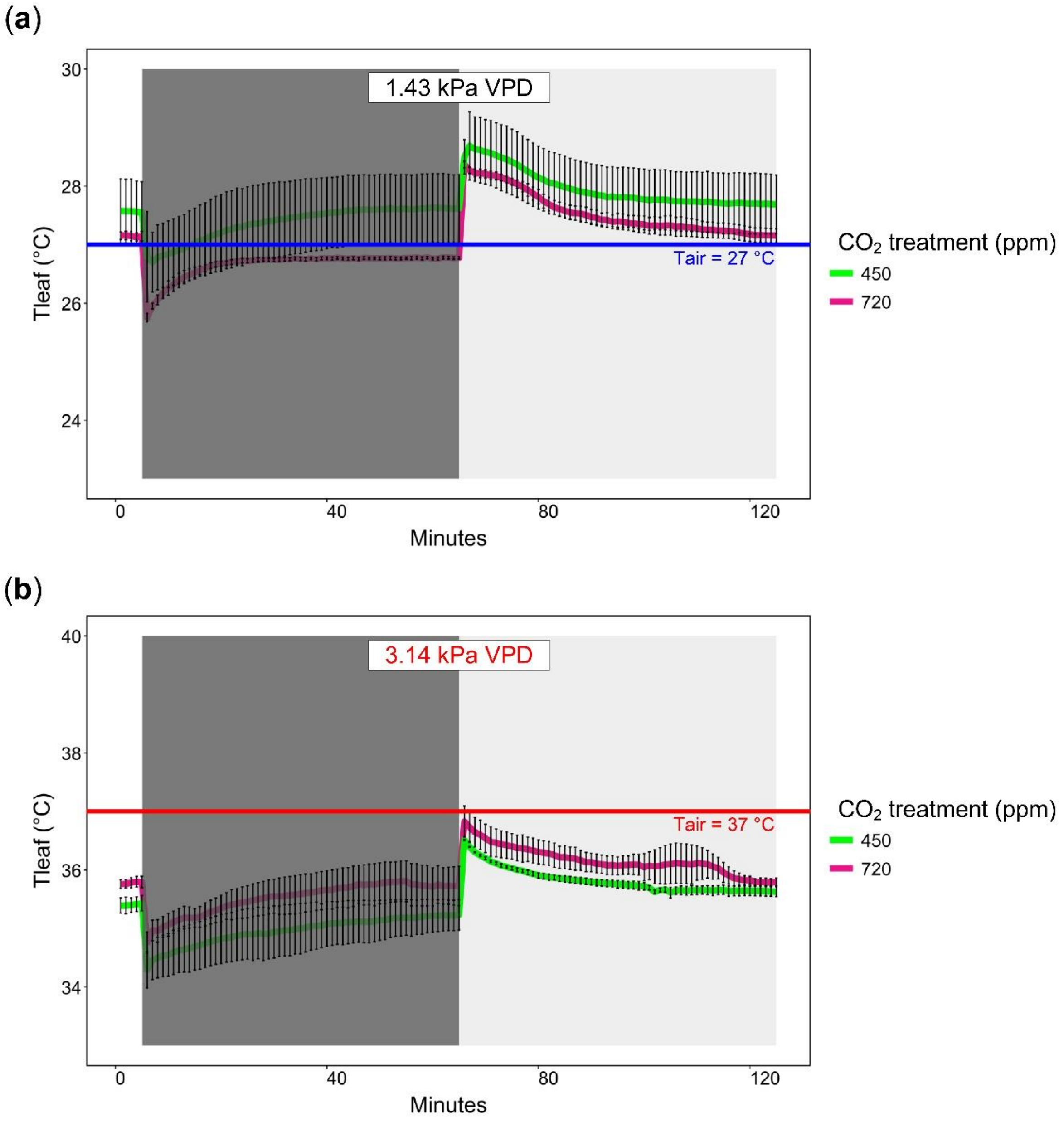

Fig. S10. Wheat leaf temperature (T_{leaf}) responses to irradiance and vapour pressure deficit (VPD) changes. (**a**) T_{leaf} measured at 1.43 VPD where T_{air} was set to 27 ºC (horizontal blue line) on plants grown and measured at either 450 ppm or 720 ppm CO_2 concentration. (**b**) T_{leaf} measured at 3.14 VPD where T_{air} was set to 37 °C (horizontal red line) on plants grown and measured at either 450 ppm or 720 ppm. Note, T_{leaf} drops by 1-1.4 °C during light switch off in both treatments and increases by 1.1-1.6 °C when light is reapplied. Whilst T_{leaf} is typically above Tair set point for ambient VPD plants, this is not the case for heatwave plants where both CO_2 treatments have considerably lower T_{leaf} values relative to T_{air}. Measurements conducted on N4 treated plants only. *n* = 3-4.

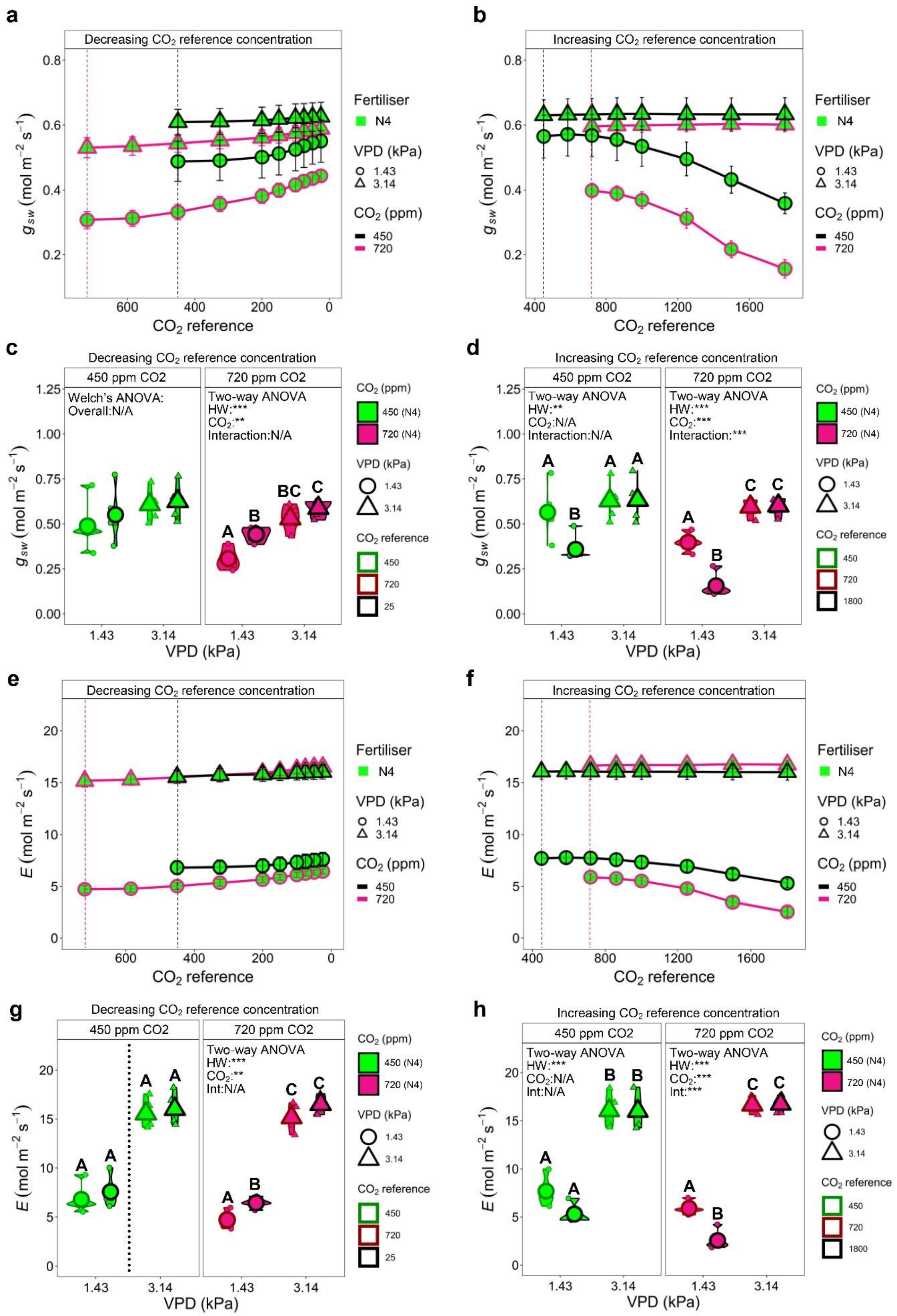

Fig. S11. Wheat stomatal conductance to water vapour (g_{sw}) and transpiration (E) responses to changes in CO_2 reference generated from CO_2 response curves. (a-b)

Assessment of g_{sw} changes during A/C_i curves from different CO_2 and vapour pressure deficit (VPD) growth scenarios. (**a**) Decreasing and (**b**) increasing CO_2 reference. (**c**-**d**) Assessment of g_{sw} differences between plants grown at 450 ppm or 720 ppm CO_2 concentration. (**c**) Decreasing CO_2 reference analysis and (**d**) increasing CO_2 reference. Comparisons were between minimum CO_2 reference (25 ppm) and growth CO_2 reference (450 or 720 ppm), or growth CO_2 reference (450 or 720 ppm) and maximum CO_2 reference analysis (1800 ppm). (**e**-**f**) Assessment of E changes during A/C_i curves from different CO_2/ and vapour pressure deficit (VPD) growth scenarios. (**e**) Decreasing and (**f**) increasing CO_2 reference. (**g**-**h**) Assessment of E differences between plants grown at 450 ppm or 720 ppm CO_2 concentration. (**g**) Decreasing CO_2 reference analysis and (**h**) increasing CO_2 reference analysis based on same CO_2 reference concentrations as in **c**-**d**. $n = 5$. Large symbols equal means. For Two-way ANOVAs, Tukey post-hoc tests were performed. For Welch's ANOVAs, Games-Howell tests were undertaken. Dotted lines in **a**, **b**, **e** and **f** highlight the starting CO_2 concentration of the curve. In **g**, the dashed line indicates two one-tailed Student's t-tests were undertaken. Different letters within graphs indicate significant differences of $p \leq 0.05$. Asterisks equal, * = $p < 0.05$, ** = $p < 0.01$ and *** = $p < 0.001$.

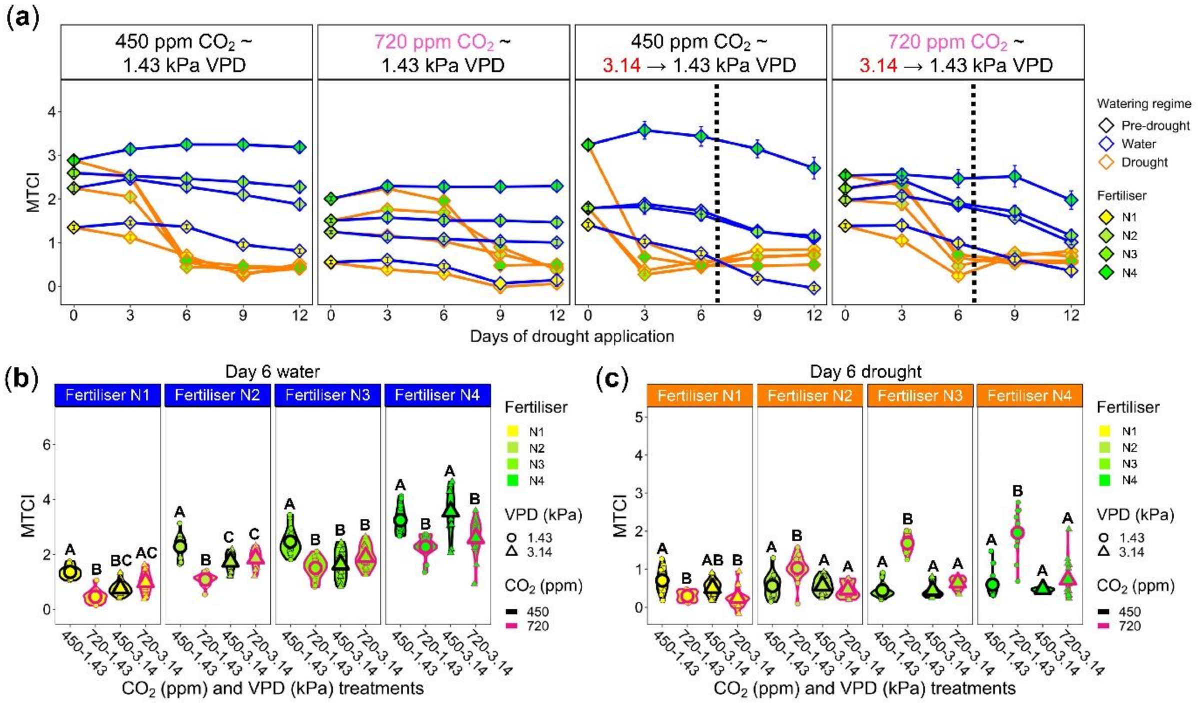

Fig. S12. Assessment of MERIS terrestrial chlorophyll index (MTCI) changes during wheat drought treatment. (**a**) MTCI plotted over at 3-day intervals over the 12 days of drought, for plants grown under the following treatments: 450 ppm CO_2, non-heatwave (vapour pressure deficit (VPD): 1.43 kPa), 720 ppm CO_2, non-heatwave, 450 ppm CO_2, heatwave (VPD 3.14 kPa) and 720 ppm CO_2 heatwave conditions. At day 7, VPD was reduced from 3.14 kPa → 1.43 kPa for heatwave plants (see dotted lines). * a total of 5 highly negative values of MTCI were removed from day 12 measurements prior to producing the above graphs (**b**-**c**) Day 6 MTCI of (**i**) continually watered plants and (**j**) droughted plants. For statistical analysis three-way generalised linear models (GLMs) were employed, with estimated marginal means computed to probe for differences within N-fertiliser groupings, with the cld function and Sidak adjustment applied to detect significance between treatments. Within individual graphs, different letters indicate significant differences of $p \leq 0.05$. See Tables S23-S24 for statistical information relating to the significance of treatments and potential interactions for three-way GLM analyses.

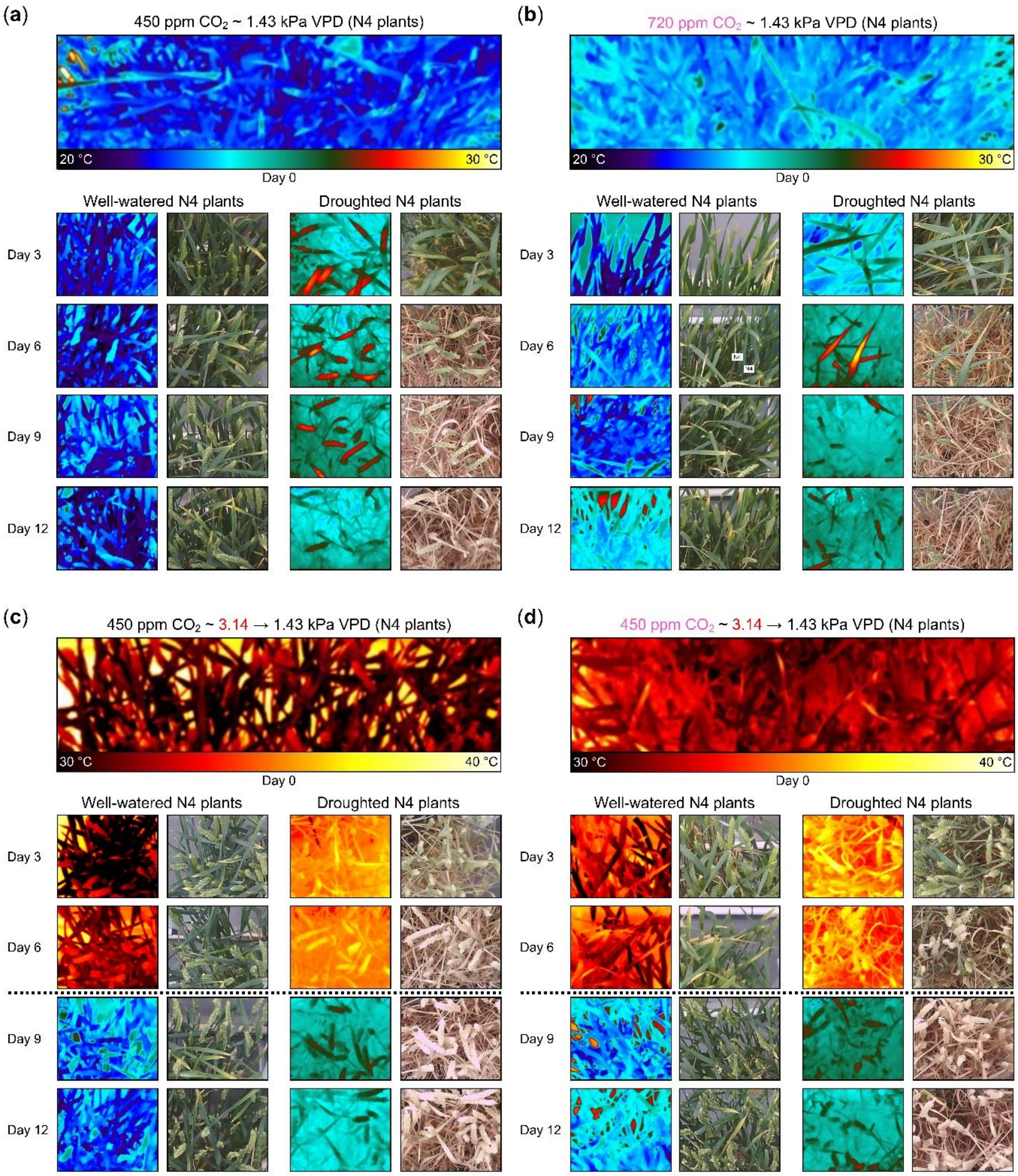

Fig. S13. Thermal profiling of nitrogen (N) fertiliser N4 wheat plants across 12 days of drought treatment. (**a-d**) Thermal and digital images of N4 plants during (**a**) 450 ppm CO_2, non-heatwave (vapour pressure deficit (VPD): 1.43 kPa), (**b**) 720 ppm CO_2, non-heatwave, (**c**) 450 ppm CO_2, heatwave (VPD 3.14 kPa) and (**d**) 720 ppm CO_2 heatwave conditions. At day 7, VPD was reduced from 3.14 kPa → 1.43 kPa for heatwave plants (see dotted lines).

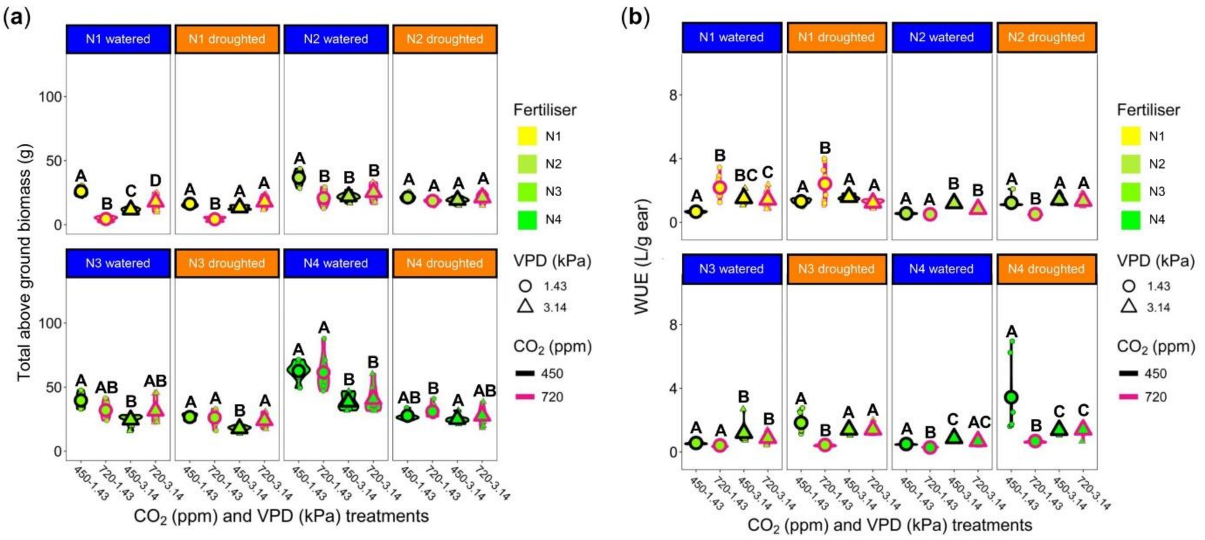

Fig. S14. Total biomass and water-use efficiency (WUE) of wheat plants grown under different CO_2, vapour pressure deficit (VPD), nitrogen (N) fertiliser regimes with continual irrigation or drought treatment. (**a**) Total above ground biomass production and (**b**) WUE. WUE calculated by dividing total water application by ear weight. $n = 8$. Large symbols equal means. For **a** and for **b**, two separate three-way generalised linear models (GLMs) were employed (one for watered, one for droughted), with estimated marginal means computed to probe for differences within N-fertiliser groupings, with the cld function and Sidak adjustment applied to detect significance between treatments. Within individual graphs, different letters indicate significant differences of $p \leq 0.05$. See Tables S27-S30 for statistical information relating to the significance of treatments and potential interactions for three-way GLM analyses.

Table S1. Summary table of 3-way ANOVA showing any individual or combined significant effects associated with vapour pressure deficit (VPD), CO_2 and/or nitrogen (N) fertiliser treatment on saturating light photosynthesis (A_{sat}) in wheat.

	Df	Sum Sq	Mean Sq	F value	Pr(>F)	
VPD	1	639.7	639.7	26.770	2.62e-06	***
CO_2	1	197.8	197.8	8.278	0.00550	**
N-Fertiliser	3	2630.9	877.0	36.699	9.23e-14	***
VPD : CO_2	1	746.5	746.5	31.240	5.44e-07	***
VPD : N-Fertiliser	3	341.1	113.7	4.758	0.00475	**
CO_2 : N-Fertiliser	3	679.5	226.5	9.478	3.05e-05	***
VPD : CO_2 : N-Fertiliser	3	21.2	7.1	0.295	0.82869	
Residuals	62	1481.5	23.9			

Signif. codes: 0 '***' 0.001 '**' 0.01 '*' 0.05 '.' 0.1 ' ' 1

Table S2. Summary table of 3-way ANOVA showing any individual or combined significant effects associated with vapour pressure deficit (VPD), CO_2 and/or nitrogen (N) fertiliser treatment on saturating light transpiration (E_{sat}) in wheat.

	Df	Sum Sq	Mean Sq	F value	Pr(>F)	
VPD	1	0.27972	0.27972	1084.461	< 2e-16	***
CO_2	1	0.03186	0.03186	123.532	< 2e-16	***
N-Fertiliser	3	0.01948	0.00649	25.171	9.00e-11	***
VPD : CO_2	1	0.00462	0.00462	17.907	7.79e-05	***
VPD : N-Fertiliser	3	0.00376	0.00125	4.853	0.00426	**
CO_2 : N-Fertiliser	3	0.01127	0.00376	14.563	2.75e-07	***
VPD : CO_2 : N-Fertiliser	3	0.00311	0.00104	4.019	0.01118	*
Residuals	62	0.01599	0.00026			

Signif. codes: 0 '***' 0.001 '**' 0.01 '*' 0.05 '.' 0.1 ' ' 1

Table S3. Summary table of 3-way ANOVA showing any individual or combined significant effects associated with vapour pressure deficit (VPD), CO_2 and/or nitrogen (N) fertiliser treatment on saturating light stomatal conductance (g_{sat}) in wheat.

	Df	Sum Sq	Mean Sq	F value	Pr(>F)	
VPD	1	0.8729	0.8729	95.122	3.89e-14	***
CO_2	1	0.8265	0.8265	90.060	1.08e-13	***
N-Fertiliser	3	0.2934	0.0978	10.656	9.63e-06	***
VPD : CO_2	1	0.0161	0.0161	1.752	0.1905	
VPD : N-Fertiliser	3	0.0545	0.0182	1.978	0.1265	
CO_2 : N-Fertiliser	3	0.0767	0.0256	2.786	0.0481	*
VPD : CO_2 : N-Fertiliser)	3	0.0668	0.0223	2.428	0.0738	.
Residuals	62	0.5690	0.0092			

Signif. codes: 0 '***' 0.001 '**' 0.01 '*' 0.05 '.' 0.1 ' ' 1

Table S4. Analysis of deviance table generated from the corresponding generalised linear model showing any individual or combined significant effects associated with vapour pressure deficit (VPD), CO_2 and/or nitrogen (N) fertiliser treatment on instantaneous water-use efficiency (IWUE) (A_{sat}/ E_{sat}) in wheat.

	LR Chisq	Df	Pr(>Chisq)	
VPD	52.669	1	3.948e-13	***
CO_2	110.776	1	< 2.2e-16	***
N-Fertiliser	1.852	3	0.60366	
VPD : CO_2	22.483	1	2.121e-06	***
VPD : N-Fertiliser	1.353	3	0.71650	
CO_2 : N-Fertiliser	9.669	3	0.02160	*
VPD : CO_2 : N-Fertiliser	6.498	3	0.08976	.

Signif. codes: 0 '***' 0.001 '**' 0.01 '*' 0.05 '.' 0.1 ' ' 1

Table S5. Summary table of 3-way ANOVA showing any individual or combined significant effects associated with vapour pressure deficit (VPD), CO_2 and/or nitrogen (N) fertiliser treatment on intrinsic water-use efficiency (iWUE; A_{sat}/g_{sat}) in wheat.

	Df	Sum Sq	Mean Sq	F value	Pr(>F)	
VPD	1	24402	24402	135.078	< 2e-16	***
CO_2	1	56603	56603	313.332	< 2e-16	***
N-Fertiliser	3	649	216	1.198	0.317825	
VPD : CO_2	1	2650	2650	14.671	0.000301	***
VPD : N-Fertiliser	3	295	98	0.544	0.654159	
CO_2 : N-Fertiliser	3	2281	760	4.208	0.008965	**
VPD : CO_2 : N-Fertiliser	3	1365	455	2.519	0.066188	.
Residuals	62	11200	181			

Signif. codes: 0 '***' 0.001 '**' 0.01 '*' 0.05 '.' 0.1 ' ' 1

Table S6. Analysis of deviance table generated from the corresponding generalised linear model showing any individual or combined significant effects associated with vapour pressure deficit (VPD), CO_2 and/or nitrogen (N) fertiliser treatment on leaf VPD in wheat.

	LR Chisq	Df	Pr(>Chisq)	
VPD	32.056	1	1.498e-08	***
CO_2	0.307	1	0.57974	
N-Fertiliser	0.087	3	0.99336	
VPD : CO_2	4.699	1	0.03018	*
VPD : N-Fertiliser	0.667	3	0.88090	
CO_2 : N-Fertiliser	0.125	3	0.98871	
VPD : CO_2 : N-Fertiliser	0.509	3	0.91688	

Signif. codes: 0 '***' 0.001 '**' 0.01 '*' 0.05 '.' 0.1 ' ' 1

Table S7. Summary table of 3-way ANOVA showing any individual or combined significant effects associated with VPD, CO_2 and/or fertiliser treatment on the ratio of intercellular CO_2 concentration (C_i): to ambient CO (C_a) outside of the leaf (C_i/C_a) in wheat.

	Df	Sum Sq	Mean Sq	F value	Pr(>F)	
VPD	1	0.09391	0.09391	59.342	1.3e-10	***
CO_2	1	0.01768	0.01768	11.170	0.00141	**
N-Fertiliser	3	0.00044	0.00015	0.094	0.96332	
VPD : CO_2	1	0.00023	0.00023	0.147	0.70293	
VPD : N-Fertiliser	3	0.00078	0.00026	0.164	0.91995	
CO_2 : N-Fertiliser	3	0.01417	0.00472	2.984	0.03793	*
VPD : CO_2 : N-Fertiliser	3	0.01015	0.00338	2.138	0.10447	
Residuals	62	0.09812	0.00158			

Signif. codes: 0 '***' 0.001 '**' 0.01 '*' 0.05 '.' 0.1 ' ' 1

Table S8. Analysis of deviance table generated from the corresponding generalised linear model showing any individual or combined significant effects associated with vapour pressure deficit (VPD), CO_2 and/or nitrogen (N) fertiliser treatment on total leaf chlorophyll by weight in wheat.

	LR Chisq	Df	Pr(>Chisq)	
VPD	1.82	1	0.1775	
CO_2	169.22	1	< 2.2e-16	***
N-Fertiliser	331.62	3	< 2.2e-16	***
VPD : CO_2	80.22	1	< 2.2e-16	***
VPD : N-Fertiliser	69.00	3	6.996e-15	***
CO_2 : N-Fertiliser	60.11	3	5.571e-13	***
VPD : CO_2 : N-Fertiliser	80.23	3	< 2.2e-16	***

Signif. codes: 0 '***' 0.001 '**' 0.01 '*' 0.05 '.' 0.1 ' ' 1

Table S9. Analysis of deviance table generated from the corresponding generalised linear model showing any individual or combined significant effects associated with vapour pressure deficit (VPD), CO_2 and/or nitrogen (N) fertiliser treatment on the maximum rate of rubisco carboxylation (V_{cmax}) in wheat.

	Df	Deviance	Resid. Df	Resid. Dev	Pr(>Chi)	
			76	654017		
VPD	1	460381	75	193636	< 2.2e-16	***
CO_2	1	44962	74	148674	4.190e-13	***
N-Fertiliser	3	66688	71	81986	< 2.2e-16	***
VPD : CO_2	1	14524	70	67462	3.785e-05	***
VPD : N-Fertiliser	3	2436	67	65026	0.41578	
CO_2 : N-Fertiliser	3	8679	64	56346	0.01738	*
VPD : CO_2 : N-Fertiliser	3	4157	61	52190	0.18245	

Signif. codes: 0 '***' 0.001 '**' 0.01 '*' 0.05 '.' 0.1 ' ' 1

Table S10. Summary table of 3-way ANOVA showing any individual or combined significant effects associated with vapour pressure deficit (VPD), CO_2 and/or nitrogen (N) fertiliser treatment on the maximum rate of electron transport (J_{max}) in wheat.

	Df	Sum Sq	Mean Sq	F value	Pr(>F)	
VPD	1	181415	181415	109.613	2.99e-15	***
CO_2	1	118111	118111	71.364	7.49e-12	***
N-Fertiliser	3	124901	41634	25.156	1.03e-10	***
VPD : CO_2	1	21525	21525	13.006	0.000627	***
VPD : N-Fertiliser	3	12247	4082	2.467	0.070608	.
CO_2 : N-Fertiliser	3	23153	7718	4.663	0.005343	**
VPD : CO_2 : N-Fertiliser	3	611	204	0.123	0.946126	
Residuals	61	100958	1655			

Signif. codes: 0 '***' 0.001 '**' 0.01 '*' 0.05 '.' 0.1 ' ' 1

Table S11. Analysis of deviance table generated from the corresponding generalised linear model showing any individual or combined significant effects associated with vapour pressure deficit (VPD), CO_2 and/or nitrogen (N) fertiliser treatment on the total leaf transpiration (E) of both leaf surfaces in wheat.

	LR Chisq	Df	Pr(>Chisq)	
VPD	387.95	1	< 2.2e-16	***
CO_2	33.34	1	7.754e-09	***
N-Fertiliser	87.66	3	< 2.2e-16	***
VPD : CO_2	20.92	1	4.781e-06	***
VPD : N-Fertiliser	35.37	3	1.015e-07	***
CO_2 : N-Fertiliser	2.86	3	0.4135	
VPD : CO_2 : N-Fertiliser	3.67	3	0.2995	

Signif. codes: 0 '***' 0.001 '**' 0.01 '*' 0.05 '.' 0.1 ' ' 1

Table S12. Analysis of deviance table generated from the corresponding generalised linear model showing any individual or combined significant effects associated with vapour pressure deficit (VPD), CO_2 and/or nitrogen (N) fertiliser treatment on abaxial leaf surface transpiration (E) in wheat.

	LR Chisq	Df	Pr(>Chisq)	
VPD	402.64	1	< 2.2e-16	***
CO_2	14.65	1	0.0001293	***
N-Fertiliser	115.36	3	< 2.2e-16	***
VPD : CO_2	5.80	1	0.0160335	*
VPD : N-Fertiliser	50.38	3	6.641e-11	***
CO_2 : N-Fertiliser	18.85	3	0.0002934	***
VPD : CO_2 : N-Fertiliser	35.23	3	1.089e-07	***

Signif. codes: 0 '***' 0.001 '**' 0.01 '*' 0.05 '.' 0.1 ' ' 1

Table S13. Analysis of deviance table generated from the corresponding generalised linear model showing any individual or combined significant effects associated with vapour pressure deficit (VPD), CO_2 and/or nitrogen (N) fertiliser treatment on adaxial leaf surface transpiration (*E*) in wheat.

	LR Chisq	Df	Pr(>Chisq)	
VPD	179.510	1	< 2.2e-16	***
CO_2	20.345	1	6.465e-06	***
N-Fertiliser	35.409	3	9.983e-08	***
VPD : CO_2	14.298	1	0.000156	***
VPD : N-Fertiliser	15.169	3	0.001678	**
CO_2 : N-Fertiliser	23.151	3	3.756e-05	***
VPD : CO_2 : N-Fertiliser	4.078	3	0.253146	

Signif. codes: 0 '***' 0.001 '**' 0.01 '*' 0.05 '.' 0.1 ' ' 1

Table S14. Analysis of deviance table generated from the corresponding generalised linear model showing any individual or combined significant effects associated with vapour pressure deficit (VPD), CO_2 and/or nitrogen (N) fertiliser treatment on the % *E* contribution from the abaxial leaf surface in wheat.

	LR Chisq	Df	Pr(>Chisq)	
VPD	80.390	1	< 2.2e-16	***
CO_2	47.221	1	6.342e-12	***
N-Fertiliser	66.440	3	2.467e-14	***
VPD : CO_2	21.184	1	4.172e-06	***
VPD : N-Fertiliser	36.075	3	7.220e-08	***
CO_2 : N-Fertiliser	4.862	3	0.18218	
VPD : CO_2 : N-Fertiliser	7.944	3	0.04718	*

Signif. codes: 0 '***' 0.001 '**' 0.01 '*' 0.05 '.' 0.1 ' ' 1

Table S15. Analysis of deviance table generated from the corresponding generalised linear model showing any individual or combined significant effects associated with vapour pressure deficit (VPD), CO_2 and/or nitrogen (N) fertiliser treatment on the total stomatal conductance to water vapour (g_{sw}) of both leaf surfaces in wheat.

	LR Chisq	Df	Pr(>Chisq)	
VPD	219.924	1	< 2.2e-16	***
CO_2	20.738	1	5.266e-06	***
N-Fertiliser	148.029	3	< 2.2e-16	***
VPD : CO_2	0.384	1	0.53549	
VPD : N-Fertiliser	72.726	3	1.113e-15	***
CO_2 : N-Fertiliser	0.117	3	0.98974	
VPD : CO_2 : N-Fertiliser	13.393	3	0.00386	**

Signif. codes: 0 '***' 0.001 '**' 0.01 '*' 0.05 '.' 0.1 ' ' 1

Table S16. Analysis of deviance table generated from the corresponding generalised linear model showing any individual or combined significant effects associated with vapour pressure deficit (VPD), CO_2 and/or nitrogen (N) fertiliser treatment on the stomatal conductance to water vapour (g_{sw}) of the abaxial leaf surface in wheat.

	LR Chisq	Df	Pr(>Chisq)	
VPD	267.128	1	< 2.2e-16	***
CO_2	8.723	1	0.003143	**
N-Fertiliser	155.698	3	< 2.2e-16	***
VPD : CO_2	0.184	1	0.668059	
VPD : N-Fertiliser	70.366	3	3.563e-15	***
CO_2 : N-Fertiliser	31.303	3	7.340e-07	***
VPD : CO_2 : N-Fertiliser	51.605	3	3.635e-11	***

Signif. codes: 0 '***' 0.001 '**' 0.01 '*' 0.05 '.' 0.1 ' ' 1

Table S17. Analysis of deviance table generated from the corresponding generalised linear model showing any individual or combined significant effects associated with vapour pressure deficit (VPD), CO_2 and/or nitrogen (N) fertiliser treatment on the stomatal conductance to water vapour (g_{sw}) of the adaxial leaf surface in wheat.

	LR Chisq	Df	Pr(>Chisq)	
VPD	99.839	1	< 2.2e-16	***
CO_2	13.062	1	0.0003014	***
N-Fertiliser	76.769	3	< 2.2e-16	***
VPD : CO_2	0.198	1	0.6564988	
VPD : N-Fertiliser	41.216	3	5.885e-09	***
CO_2 : N-Fertiliser	8.739	3	0.0329727	*
VPD : CO_2 : N-Fertiliser	2.062	3	0.5596941	

Signif. codes: 0 '***' 0.001 '**' 0.01 '*' 0.05 '.' 0.1 ' ' 1

Table S18. Analysis of deviance table generated from the corresponding generalised linear model showing any individual or combined significant effects associated with vapour pressure deficit (VPD), CO_2 and/or nitrogen (N) fertiliser treatment on the % stomatal conductance to water vapour (g_{sw}) contribution from the abaxial leaf surface in wheat.

	LR Chisq	Df	Pr(>Chisq)	
VPD	62.508	1	2.654e-15	***
CO_2	30.451	1	3.425e-08	***
N-Fertiliser	47.797	3	2.353e-10	***
VPD : CO_2	14.934	1	0.0001114	***
VPD :N- Fertiliser	24.443	3	2.019e-05	***
CO_2 : N-Fertiliser	5.502	3	0.1385025	
VPD : CO_2 : N-Fertiliser	7.846	3	0.0493105	*

Signif. codes: 0 '***' 0.001 '**' 0.01 '*' 0.05 '.' 0.1 ' ' 1

Table S19. Analysis of deviance table generated from the corresponding generalised linear model showing any individual or combined significant effects associated with vapour pressure deficit (VPD), CO_2 and/or nitrogen (N) fertiliser treatment on the Δ leaf temperature relative to the reference surface in wheat.

	LR Chisq	Df	Pr(>Chisq)	
VPD	572.12	1	< 2.2e-16	***
CO_2	25.81	1	3.768e-07	***
N-Fertiliser	130.89	3	< 2.2e-16	***
VPD : CO_2	2.49	1	0.114220	
VPD : N-Fertiliser	121.98	3	< 2.2e-16	***
CO_2 : N-Fertiliser	12.17	3	0.006827	**
VPD : CO_2 : N-Fertiliser	59.35	3	8.103e-13	***

Signif. codes: 0 '***' 0.001 '**' 0.01 '*' 0.05 '.' 0.1 ' ' 1

Table S20. Summary table of 3-way ANOVA showing any individual or combined significant effects associated with vapour pressure deficit (VPD), CO_2 and/or nitrogen (N) fertiliser treatment on water usage over the first five days of the heatwave treatment in wheat.

	Sum Sq	Df	F value	Pr(>F)	
VPD	51.012	1	394.2826	< 2.2e-16	***
CO_2	0.145	1	1.1202	0.2952	
N-Fertiliser	6.146	3	15.8349	2.725e-07	***
VPD : CO_2	10.258	1	79.2860	9.813e-12	***
VPD : N-Fertiliser	1.554	3	4.0034	0.0127	*
CO_2 : N-Fertiliser	0.501	3	1.2903	0.2884	
VPD : CO_2 : N-Fertiliser	0.586	3	1.5104	0.2238	
Residuals	6.210	48			

Signif. codes: 0 '***' 0.001 '**' 0.01 '*' 0.05 '.' 0.1 ' ' 1

Table S21 Analysis of deviance table generated from the corresponding generalised linear model showing any individual or combined significant effects associated with vapour pressure deficit (VPD), CO_2 and/or nitrogen (N) fertiliser treatment on the total transpiration (*E*) of both leaf surfaces on day 6 of the drought period for wheat plants that continually received water.

	LR Chisq	Df	Pr(>Chisq)	
VPD	8.999	1	0.002701	**
CO_2	61.131	1	5.34e-15	***
N-Fertiliser	13.874	3	0.003081	**
VPD : CO_2	9.590	1	0.001957	**
VPD : N-Fertiliser	8.039	3	0.045215	*
CO_2 : N-Fertiliser	0.455	3	0.928709	
VPD : CO_2 : N-Fertiliser	8.520	3	0.036408	*

Signif. codes: 0 '***' 0.001 '**' 0.01 '*' 0.05 '.' 0.1 ' ' 1

Table S22. Analysis of deviance table generated from the corresponding generalised linear model showing any individual or combined significant effects associated with vapour pressure deficit (VPD), CO_2 and/or nitrogen (N) fertiliser treatment on the total transpiration (*E*) of both leaf surfaces on day 6 of the drought period for wheat plants that were experiencing drought.

	LR Chisq	Df	Pr(>Chisq)	
VPD	160.479	1	< 2.2e-16	***
CO_2	22.454	1	2.153e-06	***
N-Fertiliser	181.199	3	< 2.2e-16	***
VPD : CO_2	22.975	1	1.641e-06	***
VPD : N-Fertiliser	86.516	3	< 2.2e-16	***
CO_2 : N-Fertiliser	86.363	3	< 2.2e-16	***
VPD : CO_2 : N-Fertiliser	49.020	3	1.292e-10	***

Signif. codes: 0 '***' 0.001 '**' 0.01 '*' 0.05 '.' 0.1 ' ' 1

Table S23. Analysis of deviance table generated from the corresponding generalised linear model showing any individual or combined significant effects associated with vapour pressure deficit (VPD), CO_2 and/or nitrogen (N) fertiliser treatment on the leaf MERIS terrestrial chlorophyll index (MTCI) on day 6 of drought period for wheat continually receiving water.

	LR Chisq	Df	Pr(>Chisq)	
VPD	3.920	1	0.04773	*
CO_2	16.317	1	5.358e-05	***
N-Fertiliser	6.882	3	0.07577	.
VPD : CO_2	1.046	1	0.30650	
VPD : N-Fertiliser	2.616	3	0.45473	
CO_2 : N-Fertiliser	198.402	3	< 2.2e-16	***
VPD : CO_2 : N-Fertiliser	48.159	3	1.970e-10	***

Signif. codes: 0 '***' 0.001 '**' 0.01 '*' 0.05 '.' 0.1 ' ' 1

Table S24. Analysis of deviance table generated from the corresponding generalised linear model showing any individual or combined significant effects associated with vapour pressure deficit (VPD), CO_2 and/or nitrogen (N) fertiliser treatment on the leaf MERIS terrestrial chlorophyll index (MTCI) on day 6 of the drought period for wheat experiencing drought.

	LR Chisq	Df	Pr(>Chisq)	
VPD	16.376	1	5.193e-05	***
CO_2	36.550	1	1.488e-09	***
N-Fertiliser	161.553	3	< 2.2e-16	***
VPD : CO_2	28.932	1	7.496e-08	***
VPD : N-Fertiliser	32.964	3	3.277e-07	***
CO_2 : N-Fertiliser	2.337	3	0.5055	
VPD : CO_2 : N-Fertiliser	24.948	3	1.583e-05	***

Signif. codes: 0 '***' 0.001 '**' 0.01 '*' 0.05 '.' 0.1 ' ' 1

Table S25. Analysis of deviance table generated from the corresponding generalised linear model showing any individual or combined significant effects associated with vapour pressure deficit (VPD), CO_2 and/or nitrogen (N) fertiliser treatment on total ear weight from wheat plants that were continually watered.

	LR Chisq	Df	Pr(>Chisq)	
VPD	60.774	1	6.403e-15	***
CO_2	184.061	1	< 2.2e-16	***
N-Fertiliser	59.937	3	6.063e-13	***
VPD : CO_2	124.217	1	< 2.2e-16	***
VPD : N-Fertiliser	4.277	3	0.2331	
CO_2 : N-Fertiliser	111.851	3	< 2.2e-16	***
VPD : CO_2 : N-Fertiliser	64.554	3	6.249e-14	***

Signif. codes: 0 '***' 0.001 '**' 0.01 '*' 0.05 '.' 0.1 ' ' 1

Table S26. Analysis of deviance table generated from the corresponding generalised linear model showing any individual or combined significant effects associated with vapour pressure deficit (VPD), CO_2 and/or nitrogen (N) fertiliser treatment on total ear weight from wheat plants that had received the 16-day drought.

	LR Chisq	Df	Pr(>Chisq)	
VPD	0.793	1	0.3730888	
CO_2	17.519	1	2.845e-05	***
N-Fertiliser	15.343	3	0.0015460	**
VPD : CO_2	13.922	1	0.0001906	***
VPD : N-Fertiliser	25.957	3	9.736e-06	***
CO_2 : N-Fertiliser	135.301	3	< 2.2e-16	***
VPD : CO_2 : N-Fertiliser	77.394	3	< 2.2e-16	***

Signif. codes: 0 '***' 0.001 '**' 0.01 '*' 0.05 '.' 0.1 ' ' 1

Table S27. Analysis of deviance table generated from the corresponding generalised linear model showing any individual or combined significant effects associated with vapour pressure deficit (VPD), CO_2 and/or nitrogen (N) fertiliser treatment on total biomass from wheat plants that were continually watered.

	LR Chisq	Df	Pr(>Chisq)	
VPD	46.687	1	8.327e-12	***
CO_2	210.530	1	< 2.2e-16	***
N-Fertiliser	60.526	3	4.538e-13	***
VPD : CO_2	172.810	1	< 2.2e-16	***
VPD : N-Fertiliser	5.305	3	0.1508	
CO_2 : N-Fertiliser	133.967	3	< 2.2e-16	***
VPD : CO_2 : N-Fertiliser	95.719	3	< 2.2e-16	***

Signif. codes: 0 '***' 0.001 '**' 0.01 '*' 0.05 '.' 0.1 ' ' 1

Table S28. Analysis of deviance table generated from the corresponding generalised linear model showing any individual or combined significant effects associated with vapour pressure deficit (VPD), CO_2 and/or nitrogen (N) fertiliser treatment on total biomass from wheat plants that had received a 16-day drought.

	LR Chisq	Df	Pr(>Chisq)	
VPD	2.496	1	0.11413	
CO_2	41.080	1	1.462e-10	***
N-Fertiliser	47.160	3	3.213e-10	***
VPD : CO_2	39.149	1	3.926e-10	***
VPD : N-Fertiliser	8.848	3	0.03138	*
CO_2 : N-Fertiliser	36.374	3	6.241e-08	***
VPD : CO_2 : N-Fertiliser	22.395	3	5.397e-05	***

Signif. codes: 0 '***' 0.001 '**' 0.01 '*' 0.05 '.' 0.1 ' ' 1

Table S29. Analysis of deviance table generated from the corresponding generalised linear model showing any individual or combined significant effects associated with vapour pressure deficit (VPD), CO_2 and/or nitrogen (N) fertiliser treatment on litres per gram of ear produced from wheat plants that were continually watered.

	LR Chisq	Df	Pr(>Chisq)	
VPD	34.399	1	4.489e-09	***
CO_2	69.847	1	< 2.2e-16	***
N-Fertiliser	4.710	3	0.1943	
VPD : CO_2	40.321	1	2.154e-10	***
VPD : N-Fertiliser	2.189	3	0.5342	
CO_2 : N-Fertiliser	92.241	3	< 2.2e-16	***
VPD : CO_2 : N-Fertiliser	35.824	3	8.159e-08	***

Signif. codes: 0 '***' 0.001 '**' 0.01 '*' 0.05 '.' 0.1 ' ' 1

Table S30. Analysis of deviance table generated from the corresponding generalised linear model showing any individual or combined significant effects associated with vapour pressure deficit (VPD), CO_2 and/or nitrogen (N) fertiliser treatment on litres per gram of ear produced from wheat plants that had received a 16-day drought.

	LR Chisq	Df	Pr(>Chisq)	
VPD	1.867	1	0.1718	
CO_2	19.137	1	1.217e-05	***
N-Fertiliser	75.032	3	3.566e-16	***
VPD : CO_2	19.285	1	1.126e-05	***
VPD : N-Fertiliser	40.053	3	1.038e-08	***
CO_2 : N-Fertiliser	149.530	3	< 2.2e-16	***
VPD : CO_2 : N-Fertiliser	98.328	3	< 2.2e-16	***

Signif. codes: 0 '***' 0.001 '**' 0.01 '*' 0.05 '.' 0.1 ' ' 1
